# Disease-associated mutations in a bifunctional aminoacyl-tRNA synthetase gene elicit the integrated stress response

**DOI:** 10.1016/j.jbc.2021.101203

**Published:** 2021-09-17

**Authors:** Danni Jin, Sheree A. Wek, Nathan T. Kudlapur, William A. Cantara, Marina Bakhtina, Ronald C. Wek, Karin Musier-Forsyth

**Affiliations:** 1 Department of Chemistry and Biochemistry, Center for RNA Biology, The Ohio State University, Columbus Ohio, USA; 2 Department of Biochemistry and Molecular Biology, Indiana University School of Medicine, Indianapolis Indiana, USA

**Keywords:** tRNA charging, aminoacyl-tRNA synthetases, glutamyl-prolyl-tRNA synthetase, enzyme kinetics, stress response, endoplasmic reticulum stress (ER stress), multisynthetase complex, compound heterozygous mutations, genetic disease, AIMP, ARS-complex interacting multifunctional protein, ARS, aminoacyl-tRNA synthetase, ATF4, activating transcription factor 4, CD, circular dichroism, CHOP, C/EBP-homologous protein, DTT, dithiothreitol, ERS, glutamyl-tRNA synthetase, FA, fluorescence anisotropy, GAIT, gamma interferon-activated inhibition of translation, GCN2, general control nonderepressible 2, GST, glutathione-S-transferase, ISR, integrated stress response, MALS, multiangle laser light scattering, MSC, multisynthetase complex, PERK, PKR-like endoplasmic reticulum kinase, PRS, prolyl-tRNA synthetase, SEC, size-exclusion chromatography, WT, wild-type

## Abstract

Aminoacyl-tRNA synthetases (ARSs) catalyze the charging of specific amino acids onto cognate tRNAs, an essential process for protein synthesis. Mutations in ARSs are frequently associated with a variety of human diseases. The human *EPRS1* gene encodes a bifunctional glutamyl-prolyl-tRNA synthetase (EPRS) with two catalytic cores and appended domains that contribute to nontranslational functions. In this study, we report compound heterozygous mutations in *EPRS1*, which lead to amino acid substitutions P14R and E205G in two patients with diabetes and bone diseases. While neither mutation affects tRNA binding or association of EPRS with the multisynthetase complex, E205G in the glutamyl-tRNA synthetase (ERS) region of EPRS is defective in amino acid activation and tRNA^Glu^ charging. The P14R mutation induces a conformational change and altered tRNA charging kinetics *in vitro*. We propose that the altered catalytic activity and conformational changes in the EPRS variants sensitize patient cells to stress, triggering an increased integrated stress response (ISR) that diminishes cell viability. Indeed, patient-derived cells expressing the compound heterozygous EPRS show heightened induction of the ISR, suggestive of disruptions in protein homeostasis. These results have important implications for understanding ARS-associated human disease mechanisms and development of new therapeutics.

Aminoacyl-tRNA synthetases (ARSs) are essential enzymes for protein synthesis across all domains of life, ensuring translational fidelity and maintenance of protein homeostasis by aminoacylating tRNAs with their cognate amino acids. For each amino acid, with a few exceptions in some bacteria and archaea, a specific ARS catalyzes tRNA aminoacylation in two steps: (1) amino acid activation and formation of an aminoacyl-adenylate (aa-AMP) and (2) transfer of the amino acid onto the 3′ end of tRNA (1). In addition to a catalytic domain that directs the aminoacylation or charging reaction, the majority of ARSs contain an anticodon-binding domain that interacts specifically with the cognate tRNA substrate.

Many eukaryotic ARSs have appended domains with no apparent function in tRNA charging (2). For example, human glutamyl-prolyl tRNA synthetase (EPRS) is a unique bifunctional ARS, consisting of glutamyl-tRNA synthetase (ERS) and prolyl-tRNA synthetase (PRS) catalytic domains, an appended glutathione-S-transferase-like (GST-l) domain on the N-terminus of ERS and three WHEP domains in the linker region connecting the two catalytic cores (Fig. 1*A*) (3). The evolutionary fusion between the ERS and PRS genes is thought to have occurred prior to the appearance of animals, and the emergence of a bifunctional EPRS is suggested to have a metabolic advantage over separated ERS and PRS genes (4). Human EPRS is normally localized to a cytoplasmic high-molecular-weight multisynthetase complex (MSC) that is composed of seven other ARSs and three ARS-complex interacting multifunctional proteins (AIMPs) designated as AIMP1, AIMP2, and AIMP3. The GST-l domains of EPRS, methionyl-tRNA synthetase (MetRS), AIMP2, and AIMP3 form a complex that is critical for the assembly of the MSC (5, 6). While it has been suggested that the MSC facilitates tRNA channeling and efficient translation (7, 8), others proposed that the primary function for localization of ARSs in the MSC is regulation of their nontranslational functions (9, 10, 11).

**Figure 1:**
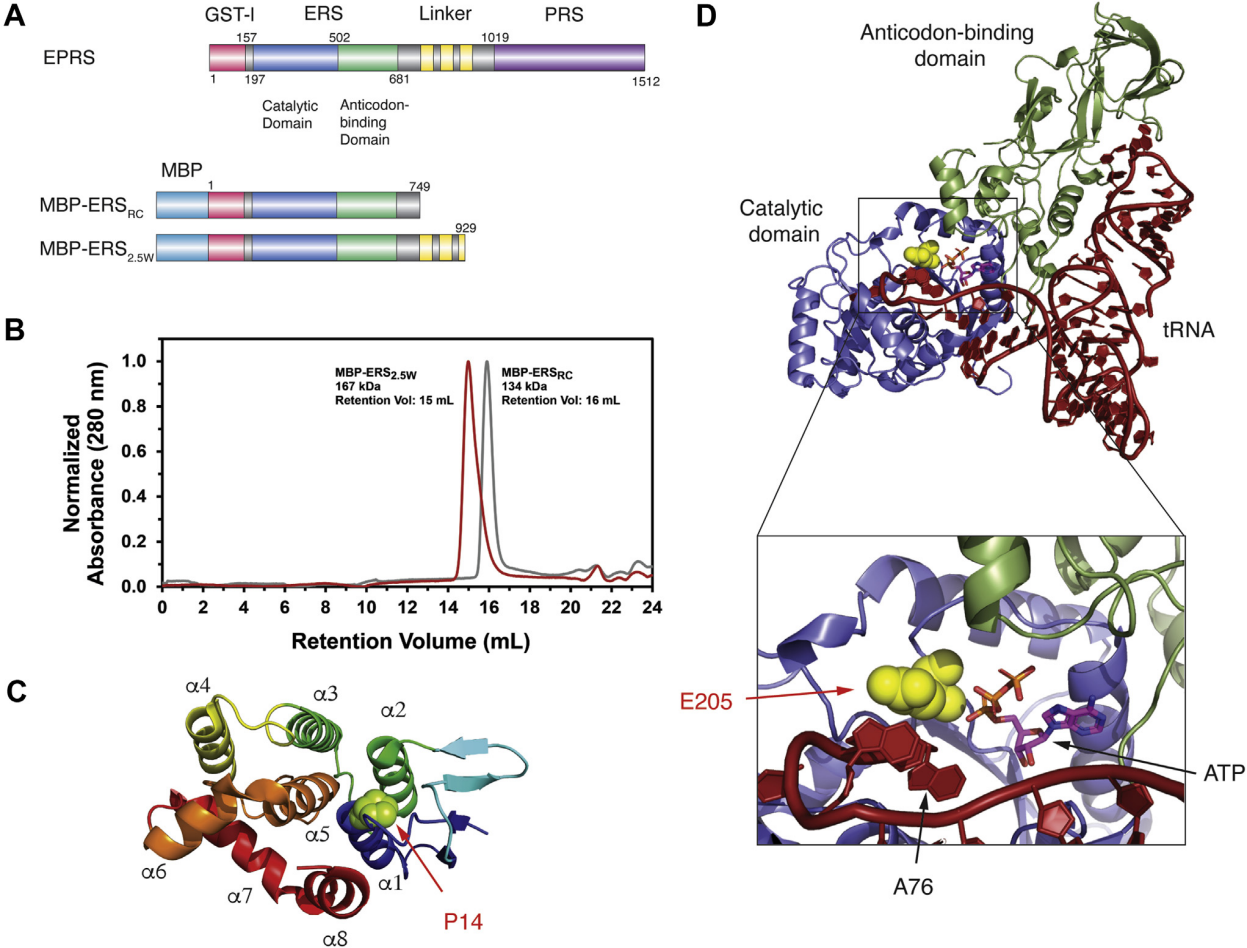
**Human EPRS constructs, location of point mutations, and SEC-MALS characterization of purified recombinant proteins.***A*, schematic of human EPRS and MBP-tagged ERS constructs purified *in vitro*. *B*, SEC-MALS chromatogram of purified WT ERS_2.5W_ and ERS_RC_. *C*, location of P14 residue in the ERS GST-l domain, adapted from Cho *et al*. (5). *D*, location of E205 in a homology model of human ERS domain bound to tRNA, based on known structure of the *E. coli* glutaminyl-tRNA synthetase-tRNA complex (PDB: 4JYZ) (64).

In addition to their role in tRNA charging, many eukaryotic ARSs have novel regulatory functions in the cell. For example, EPRS has a well-characterized role in the gamma interferon-activated inhibition of translation (GAIT) system (12, 13, 14, 15, 16), in which γ-interferon triggers phosphorylation of the linker region of EPRS, releasing it from the MSC. Free EPRS can then assemble into the GAIT complex, which inhibits the translation of inflammatory gene transcripts (reviewed in (17, 18)). Infection by RNA viruses induces another EPRS phosphorylation event that targets EPRS to an immunomodulatory pathway that inhibits viral replication (19).

Mutations in ARS genes can disrupt protein homeostasis, triggering a broad spectrum of human diseases, including neuronal disease, metabolic disorders, cancer, and autoimmune disorders (reviewed in (10, 20, 21, 22)). Several mutations in the PRS portion of human *EPRS1* gene were linked with hypomyelinating leukodystrophy, and steady-state aminoacylation assays using patient-derived lymphoblast lysate suggested reduced PRS activity compared with wild-type (WT) (23). Impaired aminoacylation of tRNAs and associated disruptions in protein homeostasis can trigger the integrated stress response (ISR) (22, 24, 25). The ensuing ISR induces expression of genes involved in protein synthesis, folding, and trafficking, which collectively serve to restore protein homeostasis. Along with key transcription factors activating transcription factor 4 (ATF4) and C/EBP-homologous protein (CHOP), the expression of EPRS and other ARSs can be increased by the ISR (25, 26, 27, 28, 29). While induction of the ISR is important for adapting to stress and restoration of protein homeostasis, excessive induction of ISR genes can instead trigger cell death (30, 31, 32).

Here, we report exome analysis of DNA derived from two Ashkenazi Jewish brothers presenting with diabetes and bone disease. Two compound heterozygous EPRS variants were identified, resulting in P14R and E205G substitutions in the N-terminal GST-l and ERS catalytic domains, respectively. To establish whether these mutations affect the canonical function of EPRS in protein synthesis, we purified WT and mutant recombinant ERS proteins and assessed their impact on enzyme structure and function *in vitro*, as well as on MSC association in cells. Sensitivity of ISR gene induction in response to stress was also evaluated in patient-derived fibroblasts expressing mutant EPRS. Our results provide mechanistic insights into patient-derived EPRS mutants and their linkage with stress sensitivity and disease.

## Results

### Identification of compound heterozygous EPRS mutations in diseased individuals

Genomic studies were performed on two Ashkenazi Jewish brothers with diabetes and bone disease. Exome analysis (33) disclosed two EPRS variants: proline 14 to arginine (p.P14R) and glutamate 205 to glycine (p.E205G) (Fig. S1). Each of the two mutations was carried by one parent, who are asymptomatic, and both are present in the patients but not in their unaffected healthy brother.

### Purification and initial characterization of recombinant human ERS proteins *in vitro*

Human EPRS is schematically shown in Figure 1*A*. To date, purification of recombinant full-length human EPRS has not been reported. However, individual ERS and PRS domains have been purified and displayed varying levels of enzymatic activity. PRS displayed robust *in vitro* amino acid activation and aminoacylation activity (34). In contrast, recombinant ERS proteins with part of the linker region were purified and showed moderate aminoacylation activity (35). In our hands, bacterial expression of an ERS-only construct containing residues 1–687 of human EPRS resulted in the protein appearing in inclusion bodies. To remedy this expression problem, we designed two maltose-binding protein (MBP)-tagged extended ERS constructs: MBP-ERS_RC_ (residues 1–749), which included amino acid sequences at the C-terminus of the ERS domain that are in a random coil (RC) region prior to the WHEP domains, and MBP-ERS_2.5W_ (residues 1–929) with the RC and 2.5 WHEP domains (Fig. 1*A*). The ERS_2.5W_ protein was previously found to be present in cells as a caspase-3 cleavage product (35). The final purified proteins were estimated to be ∼90% pure (Fig. S2*B*).

A size-exclusion chromatography–multiangle laser light scattering (SEC-MALS) analysis was performed to determine the oligomeric state of the purified ERS proteins in solution. A single peak was observed for each protein (Fig. 1*B*). Predicted molecular weights for MBP-ERS_2.5W_ and MBP-ERS_RC_ are 146 kDa and 127 kDa, respectively, which are similar to the values calculated from the SEC-MALS data (calculated values: 167.2 kDa and 134.1 kDA, Fig. 1*B*) and are indicative of monomeric states of both proteins in solution. These results are also consistent with the known monomeric state of class I synthetases (36) and with models of the MSC, where EPRS exists as a dimer only through homodimerization of the PRS domains (5, 37).

**Figure 2:**
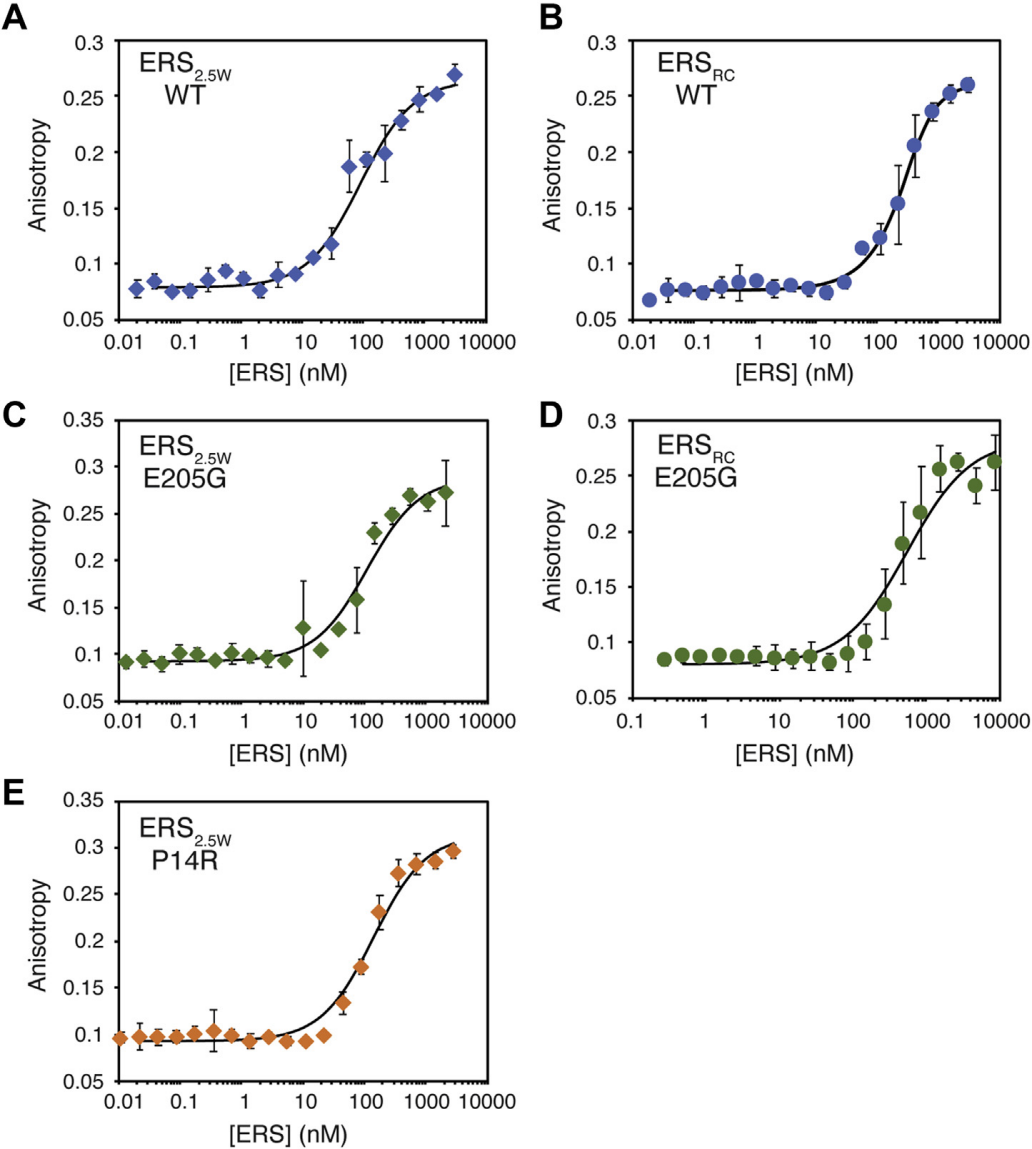
**tRNA-binding properties of recombinant ERS proteins.** Fluorescence anisotropy-binding curves of recombinant MBP-ERS proteins and *in vitro* transcribed tRNA^Glu(TTC)^. Proteins were titrated into 5 nM 3′ FTSC-labeled tRNA. (*A*) WT ERS_2.5W_; (*B*) WT ERS_RC_; (*C*) E205G ERS_2.5W_; (*D*) E205 ERS_RC_; (*E*) P14R ERS_2.5W_. Lines are fits of the data to a corrected binding isotherm described in (61). Dissociation constants (*K*_D_) listed in Table 1 were derived from three independent experiments for each recombinant protein.

We next measured the ability of purified MBP-ERS proteins to bind to tRNA using a fluorescence anisotropy (FA)-based binding assay and *in vitro* transcribed, 3′ fluorescently-labeled tRNA^Glu(TTC)^. Both ERS constructs bound tRNA^Glu^, with a *K*_d_ of 331 nM measured for MBP-ERS_RC_ and a 4-fold higher affinity (84 nM) determined for MBP-ERS_2.5W_ (Fig. 2, *A* and *B*; Table 1), suggesting that the presence of 2.5 WHEP domains facilitates tRNA binding to the ERS domain. Initial aminoacylation assays carried out under conditions of 100 nM enzyme and 4 μM tRNA showed that both ERS constructs were also active in charging unmodified, *in vitro* transcribed human tRNA^Glu^ (Fig. S3).

**Table 1:** Summary of tRNA^Glu(TTC)^-binding affinity and aminoacylation activity of MBP-ERS proteins

Protein	*K*_d_ (nM) for tRNA^Glu(TTC)^	Fold decrease in *k*_cat_/*K*_M_ of aminoacylation
MBP-ERS_2.5W_ WT	83.7 ± 41	1
MBP-ERS_2.5W_ E205G	54.3 ± 25	18
MBP-ERS_2.5W_ P14R	94.0 ± 19	1.3
MBP-ERS_RC_ WT	331 ± 120	ND
MBP-ERS_RC_ E205G	277 ± 180	ND

Abbreviation: ND, not determined.

FA-based binding assays were performed using 5 μM 3′end fluorescently-labeled tRNAs and purified MBP-ERS recombinant proteins. Aminoacylation assays were performed at 37 °C using 0.1 μM tRNA^Glu(TTC)^ and 4 nM (WT) or 10 nM (mutants) MBP-ERS proteins. Fold decreases are relative to WT MBP-ERS_2.5W_. The results are the average of three independent experiments with standard deviation listed.

### Patient-derived mutations do not affect ERS-tRNA^Glu^ binding affinity

According to a sequence alignment of higher eukaryotic EPRS (Fig. S2*A*), residues P14 and E205 are highly conserved. P14 is located in the N-terminal GST-l domain, whereas E205 is in the ERS catalytic domain, proximal to the site of ATP binding (Fig. 1, *A*, *C*, and *D*). We hypothesize that these residues are likely to play a critical function in the canonical role of EPRS in tRNA aminoacylation, and in this study we investigated the effect of these mutations on canonical ERS enzymatic activities.

Recombinant ERS proteins with P14R and E205G mutations were purified and tested for tRNA binding. As shown in Figure 2, *C* and *E* and Table 1, P14R and E205G MBP-ERS_2.5W_ bound to tRNA^Glu^ with dissociation constants of 94 nM and 54 nM, respectively, which are comparable to that of WT MBP-ERS_2.5W_ (Fig. 2*A*). Likewise, E205G MBP-ERS_RC_ displayed tRNA-binding affinity very similar to that of WT MBP-ERS_RC_ (277 nM) (Fig. 2, *B* and *D*) and approximately 5-fold lower than E205G MBP-ERS_2.5W_ (Table 1). Thus, presence of the WHEP domains facilitates tRNA binding. Taken together, these data suggest that the P14R and E205G point mutations do not significantly affect tRNA^Glu^ binding and have no influence on the facilitative role of the WHEP domains.

### E205G ERS is defective in tRNA aminoacylation

**Figure 3:**
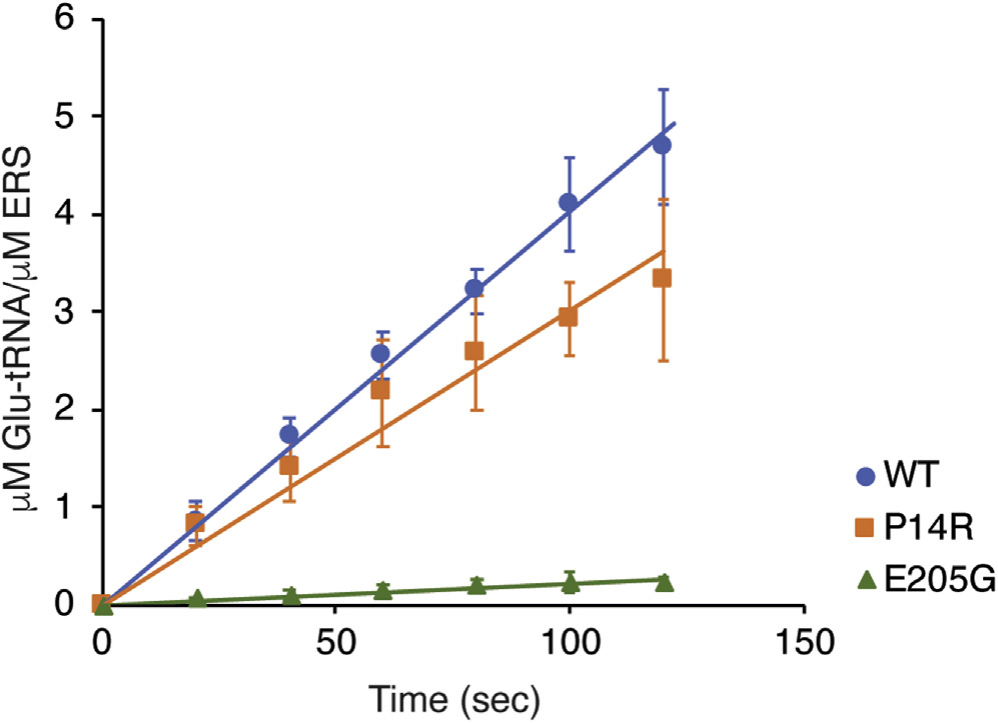
**Comparison of aminoacylation activity of WT and mutant MBP-ERS**_**2.5W**_**.** Time course of tRNA^Glu^ aminoacylation with WT and mutant MBP-ERS_2.5W_ enzymes. The reactions were performed with 0.1 μM *in vitro* transcribed tRNA^Glu(TTC)^ and 4 nM (WT) or 10 nM (mutants) MBP-ERS_2.5W_ recombinant proteins, under the condition described in Experimental Procedures.

We next investigated the aminoacylation activity of the ERS_2.5W_ mutant proteins. We first performed aminoacylation assays using low substrate tRNA^Glu^ concentrations (0.1 μM). Under these conditions, the rate of aminoacylation is directly proportional to the catalytic efficiency *k*_cat_/*K*_M_. The P14R mutant showed near WT levels of aminoacylation with a *k*_cat_/*K*_M_ value only ∼1.3-fold lower than WT (Fig. 3, Table 1). In contrast, the E205G variant had a significant 18-fold decrease in *k*_cat_/*K*_M_ compared with WT. This decrease is in agreement with the homology model showing that E205 is located near the active site and is proposed to be directly involved in ATP binding (Fig. 1*D*).

### Both P14R and E205G ERS variants display impaired amino acid activation activity

We also assessed the amino acid activation capabilities of the MBP-ERS_2.5W_ enzymes using ATP-pyrophosphate (PP_i_) exchange assays. For most tRNA-synthetases, the presence of tRNA is not required for amino acid activation. However, together with GlnRS, ArgRS, and class I LysRS, GluRS is among the exceptions (38, 39, 40). Therefore, a modified tRNA^Glu^ with a nonchargeable 3′ end is required in assays designed to isolate the amino acid activation step (41). We modified the 3′ end of *in vitro* transcribed tRNA^Glu^ by sodium periodate oxidation followed by benzylamine/sodium cyanoborohydrate stabilization steps (Fig. S4*A*) (42). The modified tRNA was not aminoacylated, as expected (Fig. S4*B*), and was used for subsequent ATP-PP_i_ exchange assays.

**Figure 4:**
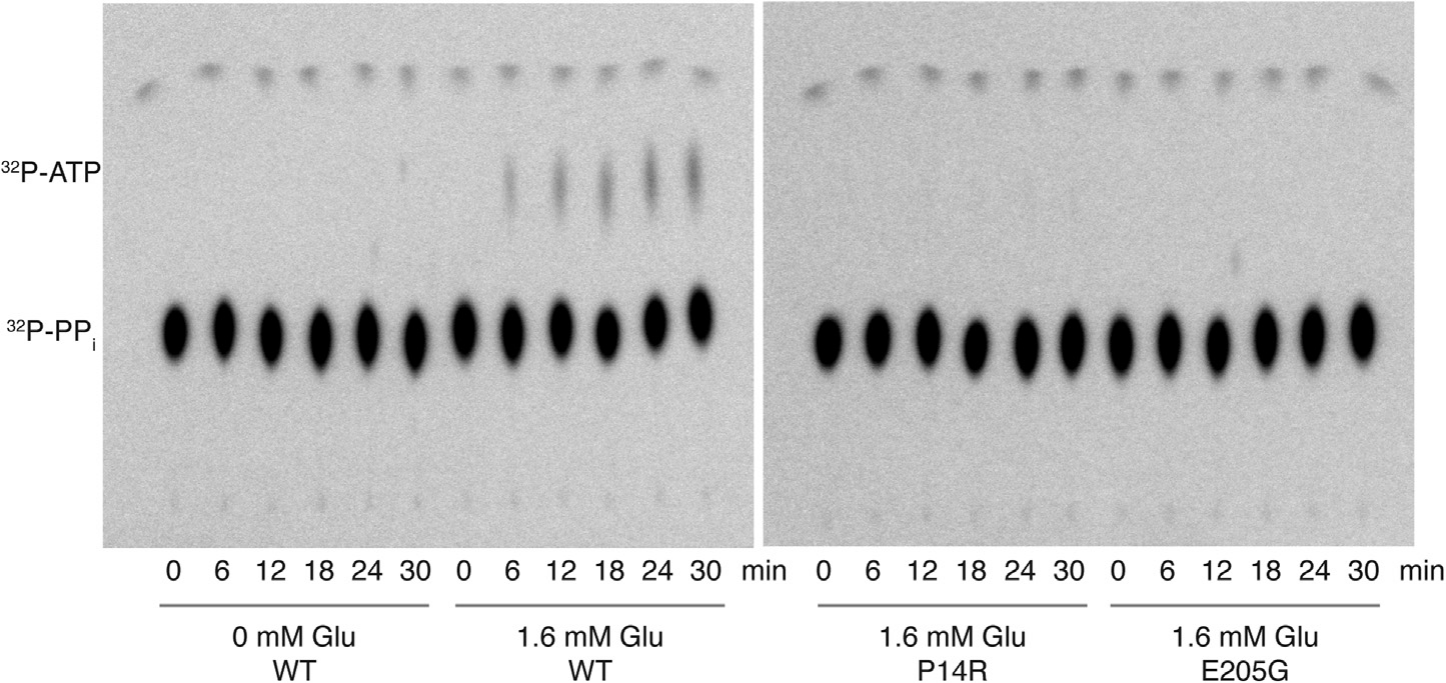
**Comparison of amino acid activation by WT and mutant ERS**_**2.5W**_. ATP-PP_i_ exchange reactions were performed using 3 μM MBP-ERS_2.5W_ recombinant proteins, 10 μM 3′ end-modified tRNA (tRNA^Glu-ox^), in the presence of ^32^P-PP_i_ and 1.6 mM glutamic acid. Reactions were separated on polyethyleneimine (PEI)-cellulose TLC plates and visualized by phosphorimaging.

WT MBP-ERS_2.5W_ displayed a low level of amino acid activation activity, as indicated by the formation of ^32^P-ATP over time (Fig. 4, left). However, no amino acid activation was detected for the P14R or E205G mutants, even in the presence of relatively high 1.6 mM Glu (Fig. 4, right). While this result is not unexpected for E205G MBP-ERS_2.5W_, which was defective in tRNA aminoacylation, the result with the P14R variant was more surprising. Taking into account that even WT showed low amino acid activation under the assay condition, the absence of detectable ^32^P-ATP formation does not necessarily mean that P14R variant completely lost the ability to catalyze glutamate activation. However, it is clear that the P14R substitution results in a significantly reduced amino acid activation rate. Based on the level of detection of this assay, we estimate that the P14R mutant is at least 10-fold defective in amino acid activation relative to the WT enzyme.

### P14R mutation causes altered ERS aminoacylation kinetics

To characterize the enzymatic activity of the P14R variant in more detail, we determined individual kinetic parameters for tRNA aminoacylation for WT and P14R MBP-ERS_2.5W_. Steady-state kinetic parameters *k*_cat_ and *K*_M_ were determined by plotting the initial rate of aminoacylation *versus* tRNA substrate concentration and fitting the plot to the Michaelis–Menten equation (Fig. S5). The results are summarized in Table 2. Interestingly, the *k*_cat_ is decreased by 8-fold in the P14R mutant compared with the WT enzyme. The *K*_M_ value has a similar fold decrease, resulting in an overall *k*_cat_/*K*_M_ that is similar to WT (Table 2), a result that is consistent with the data shown in Figure 3 and Table 1.

**Table 2:** Kinetic parameters for aminoacylation of tRNA^Glu(TTC)^ by WT and P14R MBP-ERS_2.5W_

Protein	*K*_M_ (μM)	*k*_cat_ (s^−1^)	*k*_cat_/*K*_M_ (μM^−1^ s^−1^)
MBP-ERS_2.5W_ WT	1.62 ± 0.34	0.167 ± 0.061	0.101 ± 0.021
MBP-ERS_2.5W_ P14R	0.235 ± 0.133	0.019 ± 0.006	0.095 ± 0.036

For WT enzyme, aminoacylation assays were performed with 0.5–16 μM tRNA and 100 nM protein. For P14R, 0.063–2 μM tRNA and 10 nM protein were used. The listed kinetic parameters are the averages of three independent trials with the standard deviations indicated.

To address the differences in the kinetic parameters between the WT and P14R enzymes, we performed kinetic simulation (43) of the aminoacylation reaction using an eight-step model that takes into account the requirement for tRNA binding for Glu activation (Fig. S6*A*). The aminoacylation behavior observed for the P14R mutant (*i.e.*, decrease in both *k*_cat_ and *K*_M_ values with unchanged *k*_cat_/*K*_M_) can be achieved by lowering the reaction rate of the Glu activation step for the mutant by 10-fold relative to WT (Fig. S6, *B* and *C*), which is consistent with the deleterious effect of P14R on amino acid activation that we observed in the ATP-PP_i_ exchange assays (Fig. 4). The kinetic simulation results suggest that the rate of Glu activation may become slower than the rate of Glu transfer step for the P14R enzyme, and this assumption is sufficient to explain the differences in aminoacylation kinetics observed between WT and mutant enzymes.

### P14R and E205G EPRS variants remain associated with the MSC

**Figure 5:**
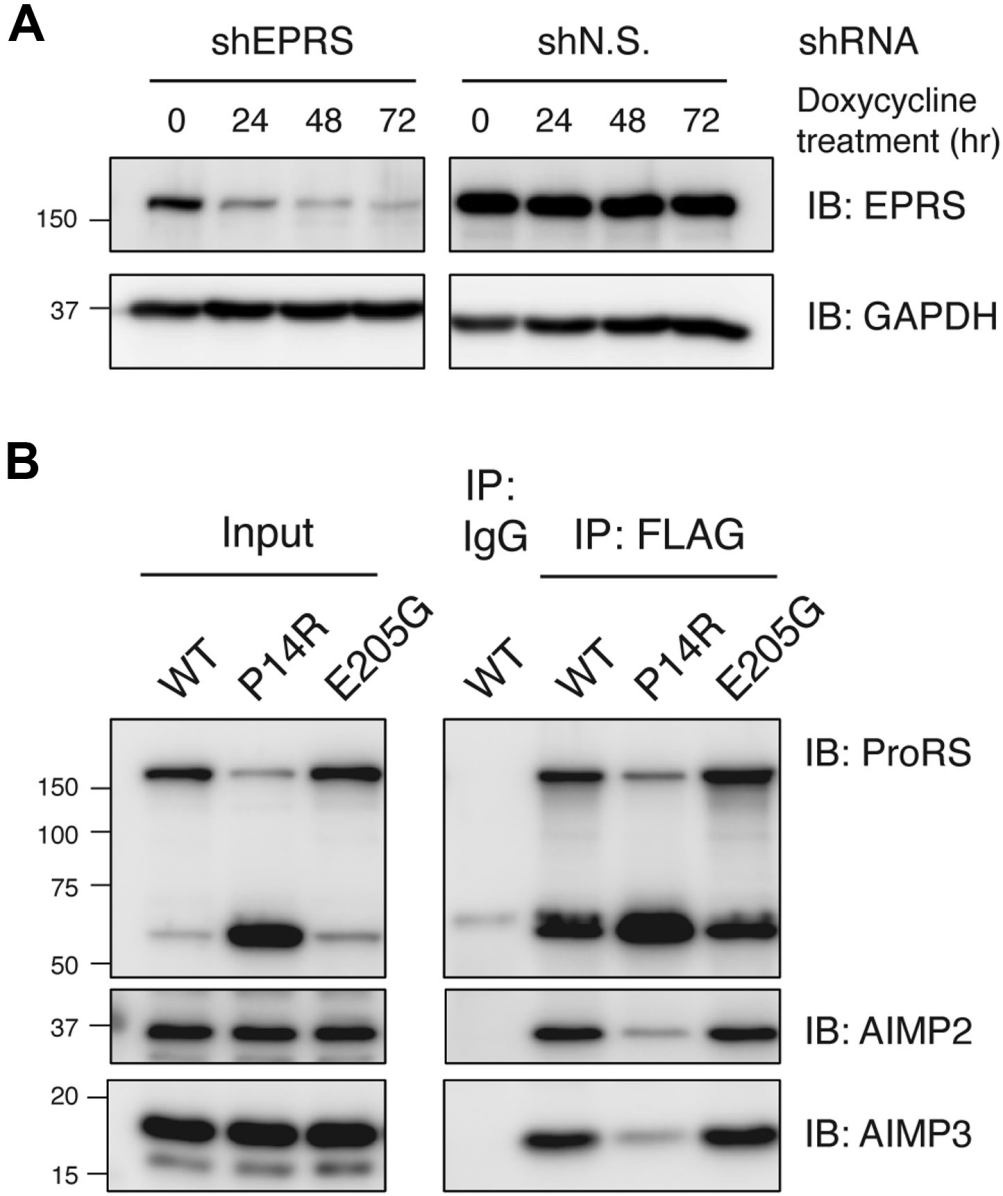
**MSC association and protein integrity of EPRS-FLAG protein overexpressed in HEK293T cells.***A*, doxycycline-inducible EPRS knockdown in HEK293T cells using shEPRS (*left*) or a nonspecific shRNA (*right*). *B*, immunoprecipitation experiments with FLAG antibody in HEK293T cells with endogenous EPRS knockdown and WT and mutant EPRS-FLAG overexpression. EPRS (*top*) and MSC scaffold proteins AIMP2 (*middle*) and AIMP3 (*bottom*) were visualized by immunoblotting using the indicated antibodies.

Given the localization of human EPRS to the cytoplasmic MSC, we tested the effect of the point mutations on EPRS association with the MSC. Within the MSC, the GST-l domain of EPRS interacts with the GST-l domains of AIMP2 and AIMP3 through two distinct protein–protein interfaces (5). A stable HEK293T cell line that allows doxycycline-inducible expression of shRNA targeting the EPRS coding sequence was generated for functional assays and displayed efficient EPRS knockdown (Fig. 5*A*). To assess whether full-length EPRS carrying the point mutations is capable of being incorporated into the MSC, we expressed C-terminal FLAG-tagged EPRS WT and mutant constructs, which are insensitive to the shRNA-derived knockdown, in HEK293T cells with endogenous EPRS knocked down and performed coimmunoprecipitation with a FLAG antibody. E205G EPRS interacts with the MSC scaffold proteins AIMP2 and AIMP3 similarly to WT (Fig. 5*B*). Interestingly, the tagged P14R EPRS expressed in HEK293T cells revealed compromised protein integrity, as evidenced by the accumulation of an ∼60 kDa truncated protein fragment (Fig. 5*B*). Lower AIMP2 and AIMP3 interaction was also detected for the P14R variant, potentially due to the loss of full-length protein. The 60 kDa fragment is derived from the C-terminal domain of EPRS, as it immunoprecipitates with the FLAG antibody and is also detected in immunoblots using a ProRS-specific antibody (Fig. 5*B*). Mass spectrometry analysis performed on the gel-extracted fragment is consistent with a polypeptide starting at position 987∼988 of human EPRS, which is in between the third WHEP domain and PRS catalytic core, and extending to the C-terminus (data not shown). The truncated form of P14R EPRS detected in HEK293T cells may be a consequence of its overexpression, as it is not present in the lysate of patient-derived cells expressing the EPRS mutants (Fig. S7). However, the loss in EPRS P14R protein integrity implies a potential conformational rearrangement in this mutant that renders the protein more susceptible to cellular proteases.

### P14R mutation induces a long-range conformational change in EPRS

**Figure 6:**
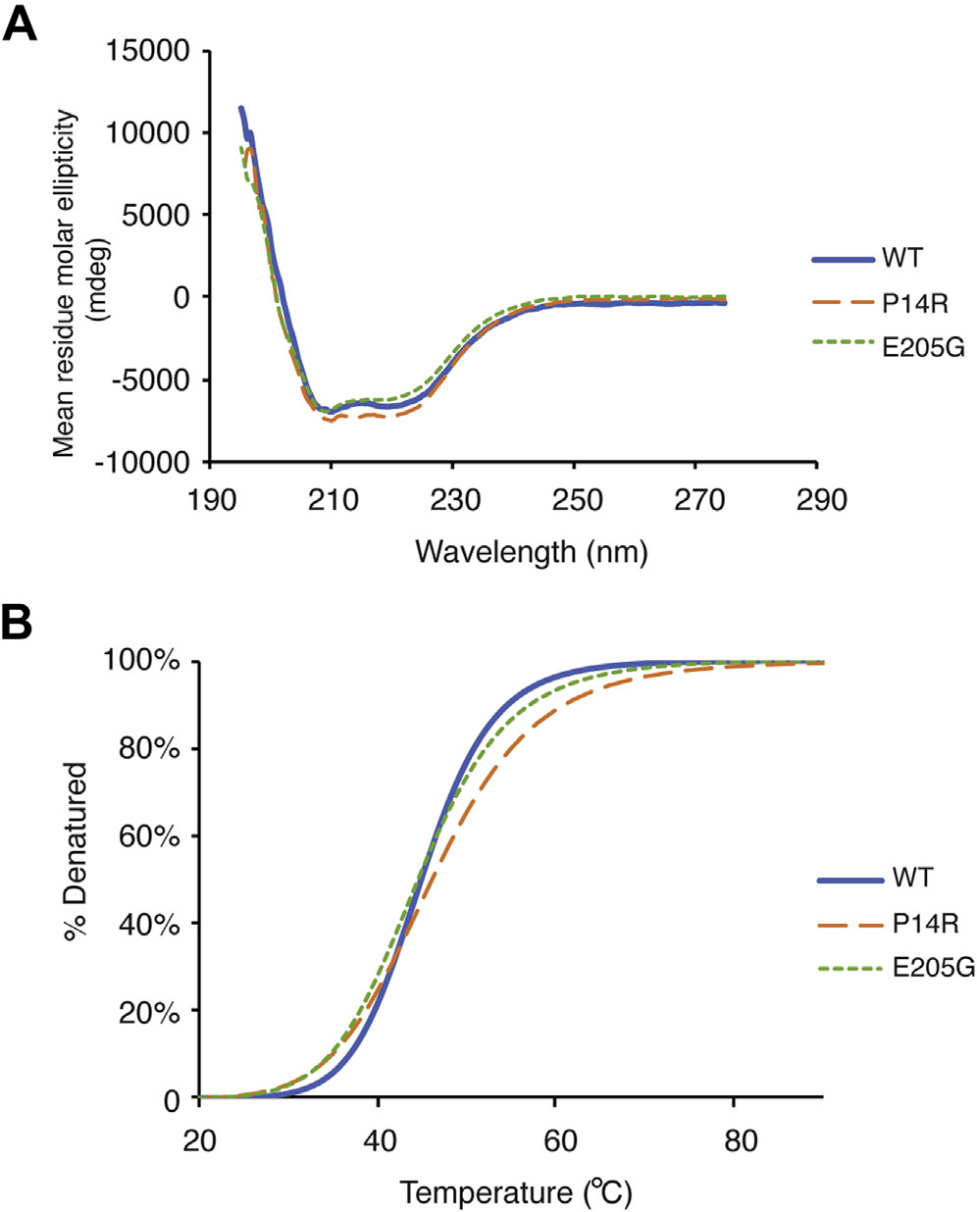
**Circular dichroism (CD) spectra and melting studies for WT and mutant MBP-ERS**_**2.5W**_**.***A*, CD spectra were collected at 20 °C and a representative spectrum of three independent trials is shown for each protein. *B*, representative melting curves generated by monitoring CD signal at 222 nm with increasing temperature. Apparent melting temperatures (T_*m*_) and Hill coefficients were derived from a fit of these curves to the Hill equation and are listed in Table 3. A representative curve of three independent trials is shown for each protein.

To gain more insights into the effect of the point mutations on ERS conformation, we used circular dichroism (CD) spectroscopy to analyze the secondary structure and thermal melting profile of WT and mutant MBP-ERS_2.5W_. At 20 °C, WT and mutant proteins displayed similar CD spectra with peak minima at 208 and 222 nm (Fig. 6*A*), suggesting an overall α-helical secondary structure. Melting studies were performed by monitoring CD signals at 222 nm as a function of increasing temperature. Data were fit to the Hill equation, and apparent melting temperatures and Hill coefficients were derived from the melting curves (Fig. 6*B*). The WT and mutant proteins have similar melting temperatures, but the two mutants had lower Hill coefficients, suggesting less cooperative melting behavior (Table 3).

**Table 3:** Apparent melting temperatures (T_*m*_) and Hill coefficients (n) for MBP-ERS_2.5W_ proteins

Protein	T_*m*_ (°C)	Hill coefficient, n
MBP-ERS_2.5W_ WT	44.7 ± 0.5	10.7 ± 0.7
MBP-ERS_2.5W_ P14R	45.4 ± 1.2	7.6 ± 0.8
MBP-ERS_2.5W_ E205G	45.2 ± 0.9	8.5 ± 0.5

Melting curves were generated by gradually heating up 3.5 μM MBP-ERS_2.5W_ protein solutions from 20 °C to 95 °C and monitoring CD signals at 222 nm. Data were fit using the Hill equation to obtain T_*m*_ and n values. The values listed are the averages of three independent trials with the standard deviations indicated.

**Figure 7:**
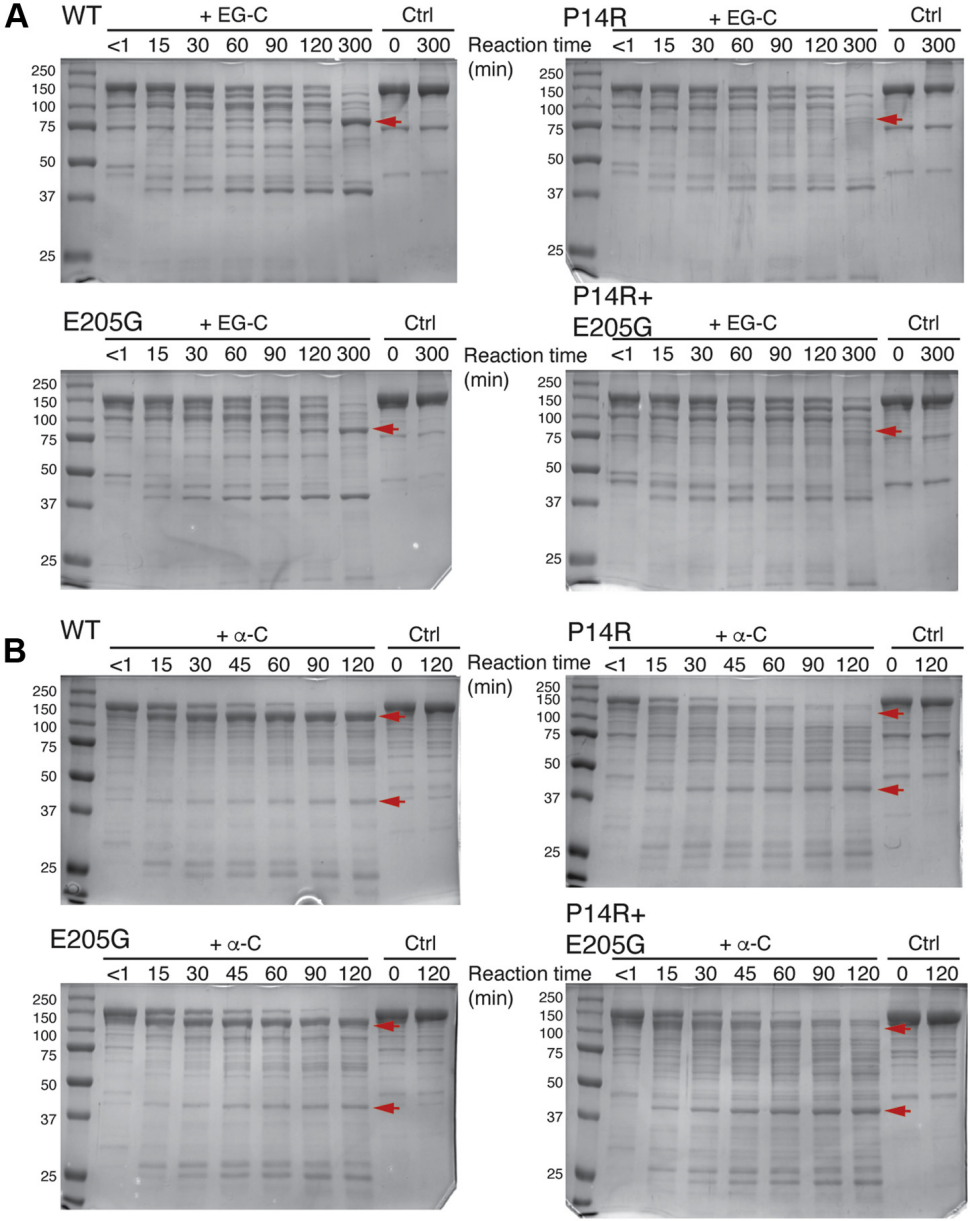
**Protease digestion patterns of WT and mutant MBP-ERS**_**2.5W**_**.** Recombinant proteins were subjected to endoproteinase Glu-C (EG-C) digestion for up to 5 h (*A*) or α-chymotrypsin (α-C) digestion for up to 2 h (*B*). Digested protein samples were analyzed by SDS-PAGE followed by Coomassie blue staining. A representative of two independent experiments is shown for each construct. *Red arrows* indicate bands with significantly altered stability in the P14R-containing mutant proteins relative to WT and E205G MBP-ERS_2.5W_.

The conformational differences between WT and mutant MBP-ERS_2.5W_ were also analyzed using a limited protease digestion assay. Recombinant proteins were treated with endoproteinase Glu-C (EG-C), which hydrolyzes peptide bonds on the C-terminus of glutamyl and aspartyl residues, or α-chymotrypsin (α-C), which cleaves peptide bonds on the C-terminus of aromatic residues. The digested samples were analyzed by SDS-PAGE. Endoproteinase Glu-C cleavage of the WT protein yielded two major fragments of approximately 80 and 40 kDa (Fig. 7*A*, top left). The E205G protein showed a similar cleavage pattern as WT (Fig. 7*A*, bottom left). In contrast, cleavage of the P14R and the double mutant did not result in the accumulation of the 80 kDa product (Fig. 7*A*, top and bottom right). WT and E205G MBP-ERS_2.5W_ also displayed a similar α-chymotrypsin digestion pattern, with a major band (doublet) appearing around 120 kDa, while P14R and double mutant proteins were digested into smaller-molecular-weight fragments over the same time course (Fig. 7*B*). Taken together, these results suggest that the P14R point mutation can induce a long-range conformational rearrangement in MBP-ERS_2.5W_, leading to altered accessibility of protease cleavage sites.

### Patient-derived cells expressing compound heterozygous EPRS mutants are sensitized for stress response

Accurate aminoacylation of tRNAs is critical for the fidelity of mRNA translation, and perturbations in these processes can disrupt protein homeostasis and trigger the ISR (24, 25). While induction of the ISR is central for alleviation of stress and restoration of protein homeostasis, high expression of certain ISR genes such as *CHOP* can instead induce maladaptive responses to stress and cell death (30, 31, 32).

**Figure 8:**
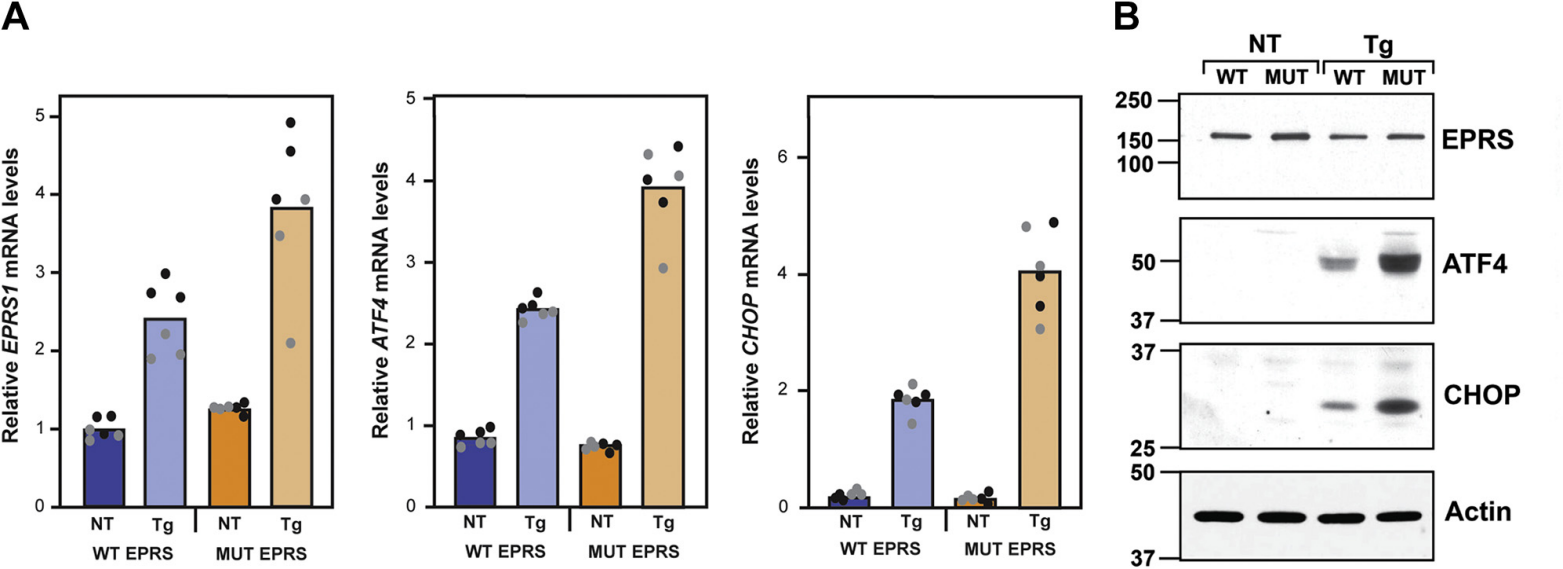
**Relative mRNA levels of *EPRS1*and ISR genes in WT and patient fibroblasts in response to ER stress.** WT EPRS and patient-derived (MUT EPRS) fibroblast cells were cultured to about 70% confluency and treated with 1 μM thapsigargin (Tg) to induce ER stress or no stress treatment (NT) for 6 h. *A*, RNA was prepared from the cells and relative levels of *EPRS1*, *ATF4*, and *CHOP* mRNAs were measured by qRT-PCR. The *black* and *gray* data points are from two experiments (indicated by *black* or *gray dots*), each with three technical replicates. *B*, protein lysates were prepared from the cells and separated by SDS-PAGE, followed by immunoblotting to detect the indicated proteins. MW markers are shown in kDa. Results are representative of two independent experiments.

To address the levels of ISR induced in patient-derived fibroblasts expressing the mutant *EPRS1* genes or WT counterparts, cells were treated with thapsigargin, a potent inducer of ER stress, and the mRNA levels of *EPRS1*, *ATF4* and *CHOP* were analyzed with real-time quantitative reverse transcription PCR (qRT-PCR) (Fig. 8*A*). In the absence of stress, patient-derived cells had similar amounts of EPRS and ISR-directed mRNAs. However, upon acute ER stress, the mutant cells showed increased mRNA levels of *EPRS* and the other ISR target genes compared with WT.

The levels of EPRS, ATF4, and CHOP proteins were also measured by immunoblot analyses in the patient-derived cells in the presence and absence of ER stress (Fig. 8*B*). *ATF4* and *CHOP* mRNAs are preferentially translated upon ER stress, and there was a more notable accumulation of ATF4 and CHOP proteins in the *EPRS* mutant cells compared with WT. Taken together, these results suggest that patient-derived cells expressing EPRS mutants are sensitized to acute stress, eliciting more ISR-directed gene expression in response to ER stress.

**Figure 9:**
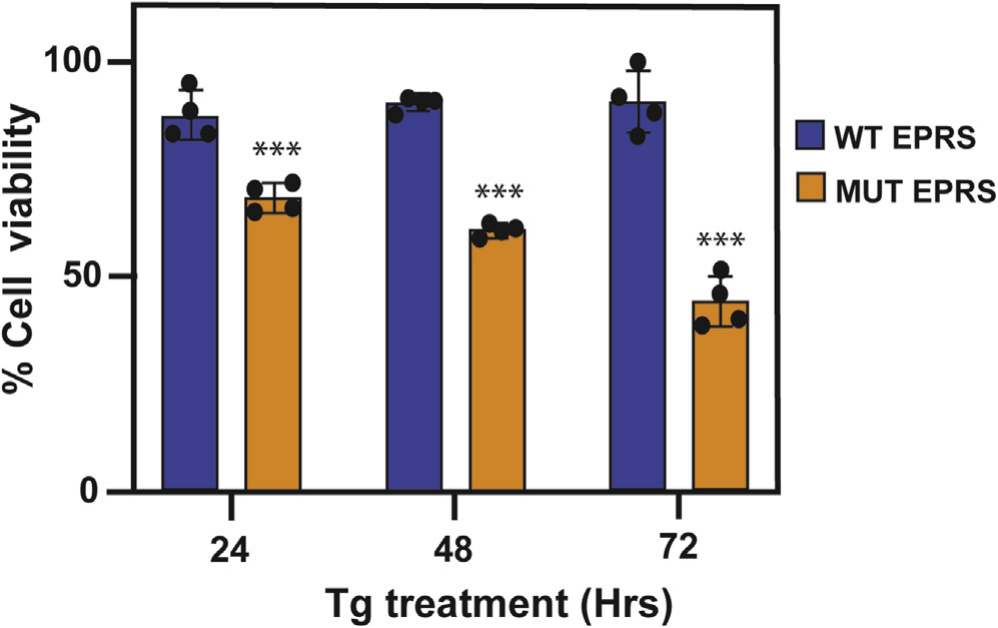
**Cell viability of WT and patient-derived cells in response to ER stress.** EPRS from WT cells or patient-derived cells with EPRS mutations (MUT EPRS) were treated with thapsigargin for up to 72 h. Cell viability was measured by the MTT assay. Values were normalized to WT nontreated cells and presented as percent cell viability. Results represent four biological replicates with data from each indicated in the bar graph. The error bars represent the SD and statistical analyses were carried out using the two-sample student *t* test. *Asterisks* indicate *p* < 0.0001.

To address whether there is a difference in the sensitivity between the WT and mutant EPRS cells, the cells were treated with thapsigargin for up to 72 h, and cell viability was evaluated by the MTT assay. While WT cells were fully resistant to the ER stress agent, the mutant EPRS cells showed progressive reductions in cell viability, with about 40% of viable cells after 72 h of thapsigargin treatment (Fig. 9). These results suggest that the catalytic dysfunctions associated with the EPRS mutants sensitize cells to stress-induced disruptions in protein synthesis, leading to sharply reduced cell viability.

## Discussion

Human ARS biallelic mutations have been associated with severe multiorgan diseases, including brain and neurological disorders, hearing and visual loss, and liver and lung diseases (44), but the association of diabetes mellitus and skeletal dysplasia was not reported. The compound heterozygous mutations encoded in the ERS domain of *EPRS* identified in two patients with diabetes and bone disease, P14R and E205G, were not previously associated with human disease, and the consequences of these mutations on ERS catalytic function *in vitro* or on protein synthesis and homeostasis *in vivo* were unknown. We successfully purified recombinant ERS proteins and showed that the E205G catalytic domain substitution results in defects in both amino acid activation and tRNA^Glu^ charging. The P14R substitution in the GST-l domain induces a protein conformational change and altered tRNA charging kinetics *in vitro*. The elevated induction of the ISR observed in patient-derived cells is consistent with disruptions in protein homeostasis. Previously, Mendes *et al.* (23) reported three missense mutations in the encoded PRS domain of EPRS that were linked with hypomyelinating leukodystrophy, a neurological disease associated with other human ARS variants. Our research suggests that missense mutations in the ERS domain contribute to biochemical changes and enhanced sensitivity to stress, and that these ERS variants may manifest themselves in the patients in different ways to present with different phenotypes than the previously reported PRS variants. It is also possible that other patient mutations independently or in combination with the ERS mutations reported here may contribute to the bone and diabetes pathologies presented by the patients.

### Purification of ERS and aminoacylation activity

While no successful purification of full-length human EPRS protein has been reported, recombinant ERS fragments of different lengths have been purified and displayed only modest levels of activity (16, 35). Whereas the MBP-ERS protein lacking any linker residues appeared in inclusion bodies (data not shown), the MBP-ERS_RC_ and MBP-ERS_2.5W_ recombinant proteins were highly soluble, suggesting that the linker region facilitates proper folding of the ERS catalytic core. Both proteins were successfully purified and displayed robust aminoacylation activity (Fig. S3). The MBP-ERS_2.5W_ construct encoding 2.5 WHEP domains displayed higher tRNA^Glu^ affinity and was therefore used in most of our subsequent *in vitro* assays. The WHEP domains were previously reported to modulate the conformation of the ERS catalytic core (35), which may lead to a conformation more optimal for tRNA binding.

The P14R ERS enzyme displayed near-WT tRNA^Glu^ aminoacylation efficiency (*k*_cat_/*K*_M_) *in vitro*, while the E205G enzyme is severely defective in charging (Table 1). Since WT *EPRS* gene is absent in the patient cells, P14R EPRS is critical for maintaining tRNA^Glu^ charging, global translation and cell viability. Interestingly, P14R ERS was saturated at lower tRNA concentration (as evidence by the reduced *K*_M_ relative to WT) but displayed a lower maximum reaction rate (as evidenced by the reduced *k*_cat_). Indeed, when we monitored aminoacylation in the presence of 4 μM tRNA^Glu^, which is saturating for both WT and P14R ERS, we observed significantly less Glu-tRNA^Glu^ formation for P14R compared with WT enzyme (Fig. S8). These results suggest that cells expressing these EPRS variants have an impaired ability to mitigate stress caused by amino acid starvation and uncharged tRNA accumulation.

### ERS amino acid activation activity

ERS is among the four ARSs that require the presence of tRNA for amino acid activation (1), which posed challenges for investigating the amino acid transfer step. While the precise mechanism by which tRNA facilitates amino acid activation by some ARSs remains unclear, it has been proposed that the 2′ OH group of the ribose of the 3′ adenosine accelerates ATP-PP_i_ exchange (45). Nevertheless, a tRNA that is incapable of being charged is required for assessing adenylate formation in these systems. Previously, adenylation measurements were achieved by performing ATP-PP_i_ exchange at low pH, conditions under which the amino acid transfer occurs slowly (46), or by using a tRNA analog with a modified 3′ adenosine (41). In this study, we modified the 3′ A76 of tRNA^Glu^ by oxidation and a subsequent stabilization reaction to disable amino acid transfer (Fig. S4). The modified tRNA used for our assay had a modified ribose ring that lacks the 2′ OH group and may not be fully capable of facilitating amino acid activation by ERS. Thus, only a low level of adenylate formation was detected for the WT enzyme (Fig. 4), and due to the limitations of the assay, only qualitative data could be obtained. Nevertheless, the mutant enzymes were both defective at this step and no product formation could be detected (Fig. 4). In the case of E205G, this is likely because of the enzyme's inability to bind ATP due to the proximity of this residue to the ATP-binding site (Fig. 1*D*). The absence of product formation for the P14R enzyme is potentially due to the low detection limit of the assay.

The P14 residue is the N-terminal cap of the proposed α-1 helix of the GST-l domain (5). Mutation of P14 to arginine could extend the helix by one residue, as residue 13 is also a proline. Previous work shows that EPRS GST-l interacts with the MSC scaffold proteins AIMP2 and AIMP3 *via* two distinct interfaces, one involving helices α-2 and α-3 and the other involving helices α-4 and α-7 (5). We show that P14R EPRS remains capable of interacting with AIMP2 and AIMP3, indicating that the EPRS-binding interfaces remain largely intact. The P14R mutation may have long-range, interdomain conformational effects on the EPRS protein, as suggested by altered ERS aminoacylation kinetics, compromised protein integrity upon EPRS overexpression in cells, and changes in the limited protease digestion pattern. We hypothesize that the P14R point mutation alters the ERS catalytic domain, slowing the amino acid activation step, making it rate-limiting for overall tRNA aminoacylation.

### ERS variants and ISR sensitivity

Expression of ARSs is increased in cells in response to stress, as a coping mechanism that results in reprogramming of global protein synthesis (27). The hallmark of the ISR pathway is the phosphorylation of eIF2α by a family of protein kinases, including general control nonderepressible 2 (GCN2) and PKR-like endoplasmic reticulum kinase (PERK). GCN2 senses and responds to amino acid deprivation by binding to uncharged tRNA (47, 48, 49) and can be activated by ARS genetic disruptions (50). PERK phosphorylates eIF2α in response to accumulation of misfolded protein causing ER stress (51). Defective mutations in PERK can result in Wolcott–Rallison syndrome, which results in diabetes (52, 53). Furthermore, both PERK and GCN2, and the downstream ISR effector ATF4, are reported to contribute to appropriate bone cell differentiation and mineralization (54, 55, 56, 57). We show here that patient cells carrying the EPRS mutations P14R and E205G are sensitized to ER stress, as evidenced by significantly elevated ATF4 and CHOP transcript and protein levels, contributing to the ISR reprogramming of the transcriptome.

Whereas the ISR provides protection for acute stress, it is suggested that inherited EPRS mutations that perturb aminoacylation of tRNA would adversely impact protein synthesis and homeostasis, eliciting chronic stress and continuous ISR induction in at least some tissues. Heightened baseline activation of the ISR in the EPRS mutant cells would reduce the stress responsive range of the ISR (32). In this way, exposure to secondary stresses such as those afflicting the ER would trigger hyperactivation of key ISR genes. Hyperaction of the ISR would enhance expression of CHOP, which would trigger cell death and tissue damage, especially in cells specialized in protein secretion, such as islet β-cells in the pancreas and those involved in bone formation. Furthermore, maintenance of ISR activation in this “adaptive zone” (32) ensures appropriate levels of eIF2α phosphorylation and translational control, which are critical for prudent regulation of transport, synthesis, and levels of free amino acids available for aminoacylation of tRNAs. While the EPRS mutations elicit the changes in aminoacylation kinetics and ISR gene expression reported here, future studies using engineered cell lines and animal models are needed to fully address the contributions of these changes to the disease phenotypes.

## Experimental procedures

### Patient whole-exome and phylogenetic analyses

Exome analysis on two Ashkenazi Jewish brothers with diabetes and bone disease was performed after informed consent using methods as previously described (33). Exonic sequences were enriched from DNA prepared from the two patients using SureSelect Human All Exon 50 Mb V.4 Kit (Agilent Technologies). Exonic sequences (100-bp paired-end) were then determined using an Illumina HiSeq2500 DNA sequencer. Alignment of the sequence read and the variant calling were carried out using the DNAnexus data analysis and management platform using the default parameters and reference human genome assembly (GRCh37/hg19). Multisequence alignments were performed using the Clustal Omega multiple sequence alignment tool. A homology model for human ERS was created based on *Escherichia coli* glutaminyl-tRNA synthetase using the ExPASy SWISS-MODEL server and edited with PyMOL software.

### Plasmid construction, protein expression, and purification

ERS-encoding genes, ERS_RC_ (aa 1–749) and ERS_2.5W_ (aa 1–929), were cloned into the pRSF vector with a small ubiquitin-like modifier (SUMO) and a maltose-binding protein (MBP) tag (a gift from Dr Kotaro Nakanishi, The Ohio State University) between *Sal*I and *Not*I restriction sites. pcDNA3 EPRS-FLAG was a gift from the laboratory of Dr Paul Fox (Cleveland Clinic). Site-directed ligase-independent mutagenesis (58, 59) was used to generate plasmids encoding P14R, E205G and double-mutant P14R + E205G-EPRS or fragments.

MBP-tagged ERS proteins were expressed in *E. coli* BL21(DE3)RIL following induction with 0.1 mM isopropyl β-D-1-thiogalactopyranoside (IPTG) at 16 °C for 24 h. Cells were lysed with lysis buffer (500 mM NaCl, 25 mM Tris-HCl pH 8, 5 mM imidazole, 5% glycerol, 3 mM 2-mercaptoethanol) containing protease inhibitor cocktail (Roche) and lysozyme (Sigma-Aldrich). Cell lysate was incubated with 0.5% v/v polyethyleneimine (PEI) to remove nucleic acids. Proteins were precipitated with 375 mg/ml ammonium sulfate and resuspended in lysis buffer. Purification was carried out using His-Select Nickel affinity chromatography (Millipore), eluting with a step gradient of imidazole. Fractions containing the protein of interest were identified by SDS–polyacrylamide gel electrophoresis (SDS-PAGE) and subjected to SUMO protease (Sigma-Aldrich) digestion according to manufacturer's instructions while dialyzing in dialysis buffer (150 mM NaCl, 25 mM Tris-HCl pH 8, 3 mM 2-mercaptoethanol) at 4° C overnight. The digested protein was further purified using size-exclusion chromatography (SEC) on a Superdex 200 16/60 column using the following elution buffer: 150 mM NaCl, 25 mM Tris-HCl pH 7.5, 1 mM dithiothreitol (DTT). Proteins were concentrated using a 30 kDa MWCO spin filter unit (Millipore) and stored at –20 °C at approximately 4 mg/ml in 12.5 mM Tris HCl pH 7.5, 75 mM NaCl, 0.5 mM DTT, and 40% glycerol. Protein concentrations were determined by BCA assay using Pierce BCA protein assay kit (Thermo Fisher).

### SEC-multiangle laser light scattering (MALS)

SEC-MALS was performed using an ӒKTA Pure 25 M chromatography system (GE Healthcare) coupled to a Dawn Helios 8+ (Wyatt) multiangle light scattering system equipped with an Optilab TrEX refractive index detector and Wyatt QELS quasi-elastic light scattering detector (Wyatt). Approximately 100 μg protein in 500 μl total volume was separated over a Superose 6 Increase column 10/300 GL (GE Healthcare) at a flow rate of 0.4 ml/min. Molecular weights were calculated using Astra 7 software based on an average of two independent replicates.

### *In vitro* transcription of tRNA and fluorescent labeling

A plasmid encoding human tRNA^Glu(TTC)^ under a T7 promotor and with a *Fok*I restriction site on the 3′ end was purchased from Integrated DNA Technologies. The tRNA was prepared *via in vitro* transcription with T7 RNA polymerase as previously described (60). The *in vitro* transcribed RNA was purified on a urea-polyacrylamide gel, eluted with RNA elution buffer (0.5 mM NH_4_OAc, 1 mM EDTA), and further isolated through butanol extraction and ethanol precipitation. The tRNA was labeled with fluorescein-5-thiosemicarbazide (FTSC) at the 3′ end as described (61).

### Fluorescence anisotropy (FA)-binding assays

The 3′ FTSC-labeled tRNA^Glu(TTC)^ was folded in 50 mM Tris-HCl pH 8, by heating at 80 °C for 2 min, 60 °C for 2 min, adding 1 mM MgCl_2_, and incubating for 5 min at room temperature followed by incubation on ice for a minimum of 30 min. The FA-binding assays were carried out as previously described (61). Fluorescently labeled tRNA (5 nM) was incubated with serially diluted MBP-ERS recombinant proteins at room temperature for 30 min in 25 mM Tris-HCl pH 8, 50 mM NaCl, and 1 mM MgCl_2_ prior to measuring FA and total intensity. Data were analyzed as described (61), and *K*_*d*_ values were derived from three independent experiments.

### Aminoacylation assays

Aminoacylation assays were performed in 20 mM Tris-HCl pH 7.5, 20 mM KCl, 10 mM MgCl_2_, 0.1 mg/ml bovine serum albumin (BSA), 4 mM DTT, 4 mM ATP, 20 μM glutamic acid, 0.3 μCi/μl [^3^H]-glutamic acid (PerkinElmer), and variable amounts of *in vitro* transcribed human tRNA^Glu(TTC)^ and MBP-ERS proteins, as indicated in the table and figure legends. Reactions were initiated by addition of MBP-ERS protein to a reaction cocktail containing folded tRNA and other components described above. Reactions were quenched on Whatman 3 MM filter pads presoaked with 5% trichloroacetic acid (TCA). The filter pads were washed three times in excess 5% TCA and one time with 95% ethanol and then dried before counting in a liquid scintillation counter (Beckman Coulter LS 6500). Each assay was performed in triplicate, and kinetic parameters were determined by fitting the data to the Michaelis–Menten equation (see Fig. S5).

### tRNA 3′ end modification and ATP-pyrophosphate (PP_i_) exchange

To prepare unchargeable tRNA, the 3′ end of *in vitro* transcribed tRNA^Glu(TTC)^ was oxidized with sodium periodate (NaIO_4_) and quenched with glycerol as described (61). The oxidized tRNA was stabilized by reacting with 20-fold molar excess of benzylamine in 20 mM Tris-HCl, pH 6.3 at room temperature for 60 min with intermittent vortexing, then reacting with 20-fold molar excess of sodium cyanoborohydride at room temperature for 60 min with intermittent vortexing. The RNA was purified with a Roche G-25 spin column and ethanol precipitation to yield the 3′-end modified tRNA^Glu-ox^ product.

For ATP-PP_i_ exchange assays, tRNA^Glu-ox^ was folded as described above and preincubated with MBP-ERS_2.5W_ proteins at room temperature for 30 min. Reactions were performed in 20 mM Tris-HCl pH 7.5, 20 mM KCl, 10 mM MgCl2, 4 mM DTT, 0.1 mg/ml BSA, 4 mM ATP, 2 mM PP_i_ and [^32^P]-PP_i_, 10 μM tRNA^Glu-ox^, 3 μM MBP-ERS_2.5W_ enzyme, and 1.6 mM glutamic acid at 37 °C. Time points were taken every 6 min over a 30 min time course by quenching 2 μl of reaction into 8 μl of 200 mM NaOAc, pH 5. Quenched reactions were spotted on PEI-cellulose TLC plates prewashed with water and methanol, then developed in 750 mM KH_2_PO_4_, pH 3.5 with 4 M urea. TLC plates were exposed to autoradiography screens (GE Healthcare) before visualization by phosphorimaging with a Typhoon Scanner (GE Healthcare).

### Circular dichroism (CD) spectroscopy

CD spectra were obtained using a Jasco J-815 spectrometer. MBP-ERS_2.5W_ proteins were buffer-exchanged into 50 mM sodium phosphate, pH 7.8, diluted to 3.5 μM (∼0.5 mg/ml), and loaded into quartz cuvettes with 1 mm pathlength. CD spectra were recorded from 200 to 275 nm at a scanning speed of 100 nm/min. Mean residue molar ellipticity was calculated as previously described (62). For thermal melting experiments, the CD signal at 222 nm was monitored at 20–90 °C temperature gradient with 2 °C increments. Melting data were fit to the Hill equation ($\mathrm{CD}={\mathrm{CD}}_{\min}+\frac{\left({\mathrm{CD}}_{\max}-{\mathrm{CD}}_{\min}\right)\times T^{n}}{{T_{m}}^{n}+T^{n}}$ , where CD_max_ and CD_min_ are CD signals at melted and folded states, respectively). The apparent melting temperature, T_*m*_, and Hill coefficient, n, were derived from the melting curves.

### Limited-protease digestion

Endoproteinase Glu-C and α-chymotrypsin used for protease cleavage experiments were from Proti-Ace Kit (Hampton Research HR2-429) and were reconstituted following the manufacturer's protocol. The proteases were diluted to 4 μg/ml in 10 mM HEPES, pH 7.5, 500 mM NaCl. To initiate the reactions, MBP-ERS_2.5W_ proteins under storage condition (4 mg/ml in 12.5 mM Tris-HCl pH 7.5, 75 mM NaCl, 0.5 mM DTT, and 40% glycerol) were mixed with an equal volume of 4 μg/ml protease solutions following a 5-min preincubation at 37 °C. The reactions were incubated at 37 °C for various time periods from 15 min to 5 h and were quenched into SDS-PAGE protein loading buffer. Cleaved protein samples (4 μg) were analyzed by SDS-PAGE.

### Cell culture and stable EPRS-knockdown cell line production

HEK293T cells were cultured in Dulbecco's modified Eagle medium (DMEM) supplemented with 10% (v/v) fetal bovine serum (FBS), 100 IU/ml penicillin, and 100 μg/ml streptomycin (complete DMEM). Patient and WT fibroblast cells were cultured in DMEM supplemented with 10% FBS. pTRIPZ plasmids encoding a doxycycline-inducible human EPRS-specific shRNA (clone ID V3THS_396942, sequence 5′-AGTTGTATAGTCTCCTCCT-3′, hereafter referred to as shEPRS) or a proprietary nonsilencing shRNA (hereafter referred to as shN.S.) were purchased from Dharmacon and were used to generate stable shRNA-expressing HEK293T cell lines by lentivirus transduction according to the manufacturer's protocol. Stable cell lines were selected and maintained in complete DMEM containing 1 μg/ml puromycin. To induce shRNA expression, cells were treated with 1.5 μg/ml doxycycline for up to 72 h.

### Immunoprecipitation and western blotting

For assessing mutant EPRS association with MSC scaffold proteins, 1.5 × 10^6^ of HEK293T shEPRS-inducible stable cells were seeded on 10 cm dishes and shRNA expression was induced with doxycycline the following day. Cells were transfected with 10 μg of pcDNA3 EPRS-FLAG (WT or mutant), using the PEI method (63) 24 h post doxycycline treatment. Cells were harvested 48 h post transfection and lysed in cell lysis buffer (Cell Signaling Technology) supplemented with protease inhibitor cocktail (Sigma-Aldrich).

For immunoprecipitation studies, 5 μg FLAG M2 mouse monoclonal antibody (Sigma-Aldrich F1804) or mouse IgG isotype control (Invitrogen 10400C) was conjugated to 25 μl Dynabeads protein G (Invitrogen) by incubating in 200 μl phosphate buffered saline with 0.01% Tween-20 (0.01% PBS-T) at room temperature for 10 min. Immunoprecipitation was performed by incubating 250 μl lysates (∼1200 μg protein) with antibody-conjugated beads overnight at 4 °C. Beads were washed three times with 0.02% PBS-T and boiled in SDS-PAGE loading buffer, followed by immunoblotting with the following antibodies: EPRS (Novus Biologicals NBP1-84929), AIMP2 (Thermo Scientific PA5-31306), AIMP3 (Invitrogen PA5-28283), and glyceraldehyde-3-phosphate dehydrogenase (GAPDH) (Bio-Rad). Immunoblots were developed for chemiluminescence and were imaged on an Amersham Imager 680 (Cytiva).

### Measuring ISR and EPRS gene expression in fibroblasts

Patient-derived fibroblasts and WT fibroblasts, derived from an unrelated individual, were cultured in DMEM supplemented with 10% FBS to about 70% confluency and then treated with 1 μM thapsigargin or no stress treatment for 6 h. Protein lysates were prepared from the cells and separated by SDS-PAGE, followed by immunoblotting using the following antibodies: EPRS (Abcam ab31531), CHOP (Santa Cruz sc-7351 and Abcam ab11419), ATF4 (purified custom antibody), and β-actin (Sigma-Aldrich A5441). RNAs were prepared from the treated cells and relative levels of *EPRS*, *ATF4*, and *CHOP* mRNAs were measured by real-time quantitative reverse transcription PCR (qRT-PCR) with the following primers: CHOP (forward: 5′ AGCCAAAATCAGAGCTGGAA 3′, reverse: 5′ ACAAGTTGGCAAGCTGGTCT 3′); ATF4 (forward: 5′ TCAAACCTCATGGGTTCTCC 3′, reverse: 5′ GTGTCATCCAACGTGGTCAG 3′); EPRS (forward: 5′ GCAGGCCTTCCAGTCAGTAG 3′, reverse: 5′ TGCATGCCCAATGTGTAAGT 3′), Actin (forward: 5′ GGACTTCGAGCAAGAGATGG 3′, reverse: 5′ AGCACTGTGTTGGCGTACAG 3′).

### MTT assays

Equal amounts of WT and patient-derived EPRS mutant cells were seeded in DMEM supplemented with 10% FBS in 96-well culture plates at 5000 cells/well. The following day, cells were treated with 1 μM thapsigargin for up to 72 h or no stress treatment. Cell viability was assessed through the conversion of tetrazolium (MTT) to formazan by measuring the absorbance at 570 nm using the CellTiter 96-well nonradioactive cell proliferation assay (Promega, #G4000). Absorbance values were normalized to WT nontreated cells and presented as cell viability.

## Data availability

All data are contained within the manuscript.

## Supporting information

This article contains supporting information (42).

## Conflict of interest

R. C. W. serves as a scientific advisor to HiberCell. All other authors declare that they have no conflicts of interest with the contents of this article.
