# Supplementary material for: Disease-associated mutations in a bifunctional aminoacyl-tRNA synthetase gene elicit the integrated stress response
Source: J Biol Chem. 2021 Sep 17;297(4):101203. doi: 10.1016/j.jbc.2021.101203 (PMC8511952; doi:10.1016/j.jbc.2021.101203)
Supplement: Figures S1–S8 [file mmc1.docx]

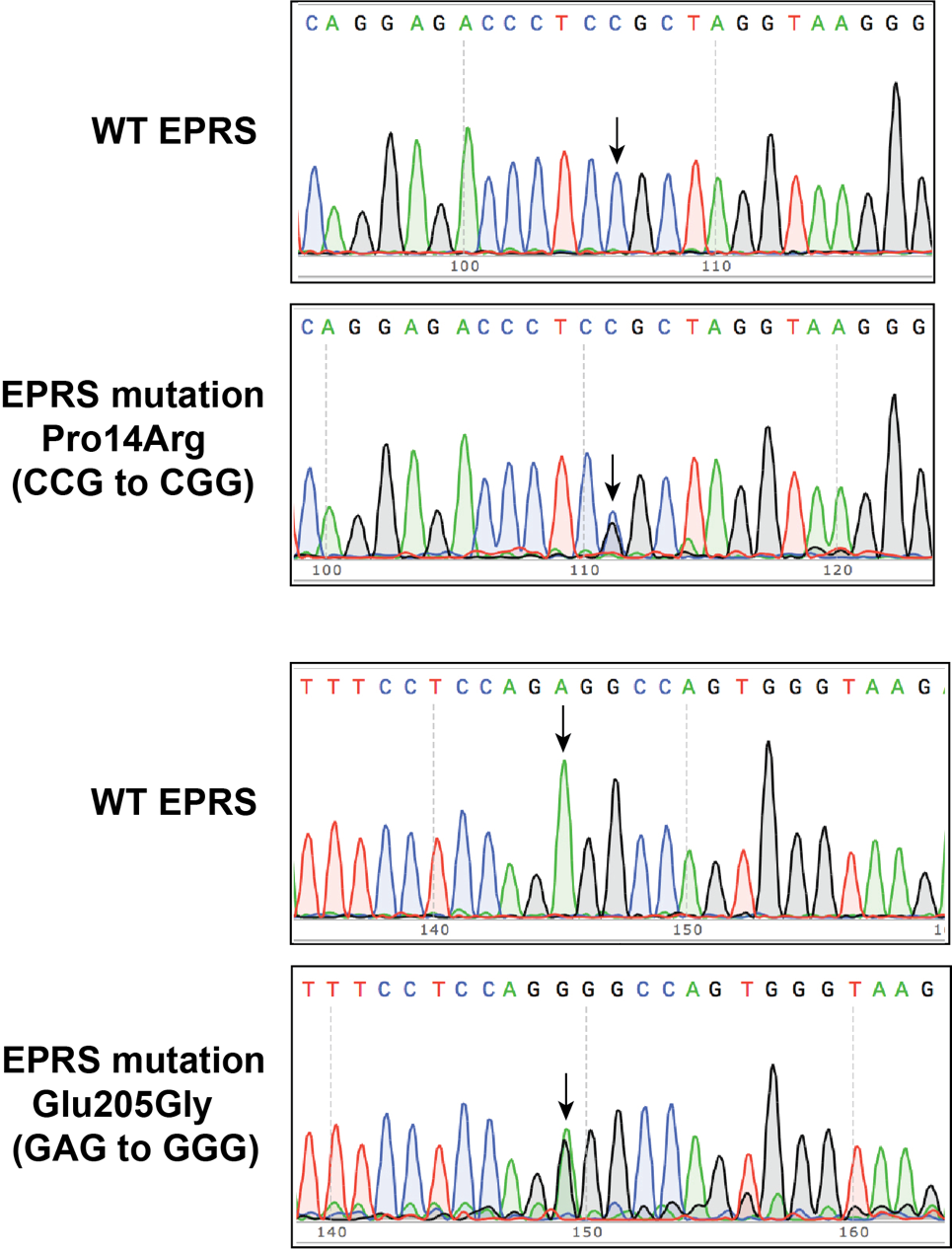


**Figure S1. Identification of compound heterozygous EPRS mutations P14R and E205G in patient-derived *EPRS1* genes.** Mutations in a patient-derived *EPRS1* gene indicate single nucleotide changes that result in amino acid substitutions Pro14Arg and Glu205Gly. Regions of the *EPRS1* gene were amplified from genomic DNA prepared from fibroblast cells prepared from the patient and normal reference (WT). DNA sequences were determined by the dideoxy method and the resulting chromatograms are illustrated. The arrows indicate base changes in the patient who is a compound heterozygote.

**
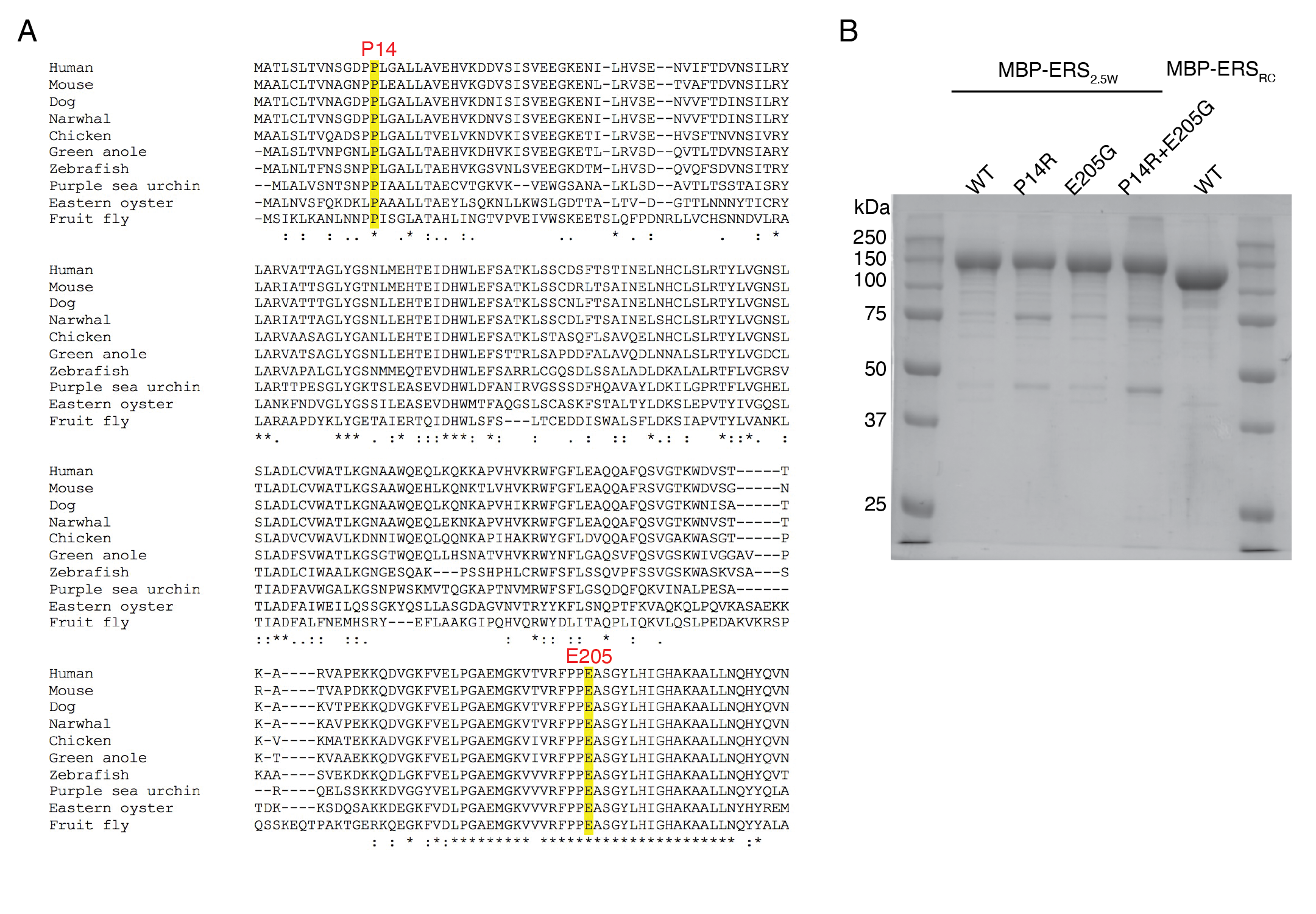
**

| Figure S2. (A) Alignment of EPRS sequences from higher eukaryotes with the position of the patient-derived amino acid changes highlighted in yellow. (B) Purified recombinant ERS proteins analyzed by SDS-PAGE and Coomassie blue staining. |
| --- |

**
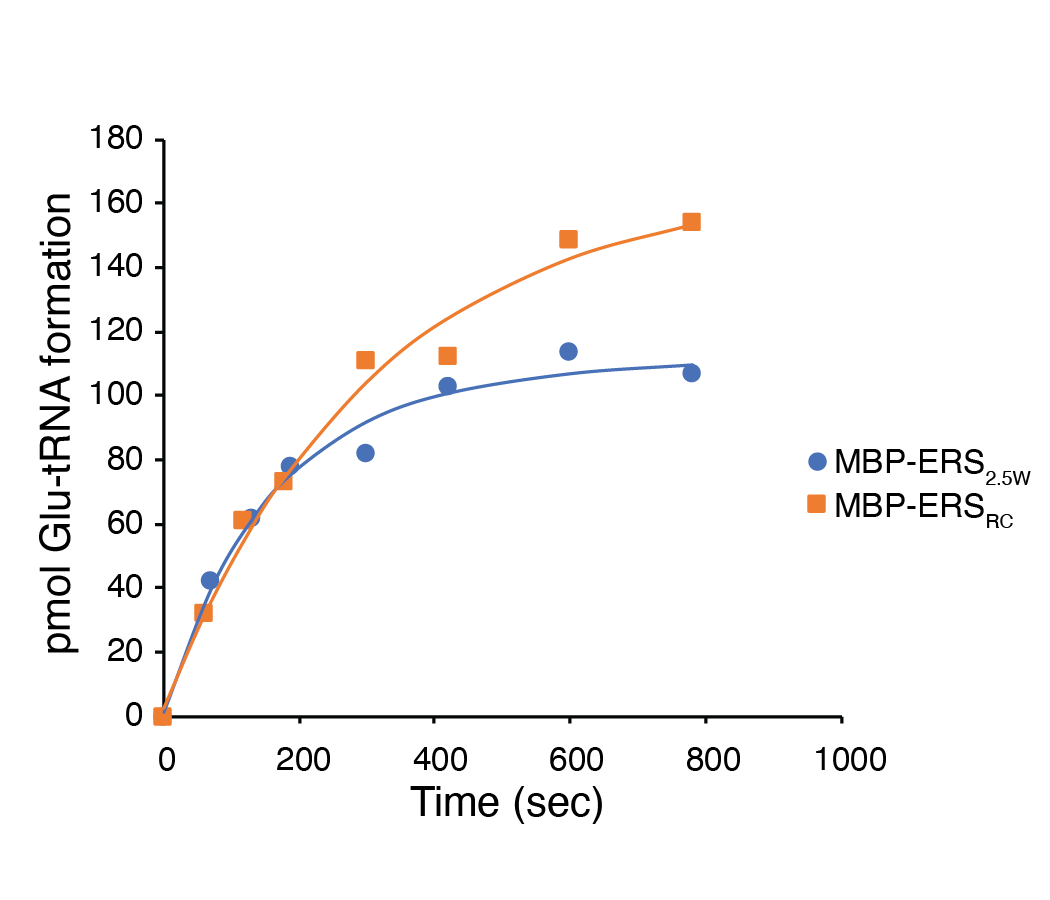
**

| Figure S3. Aminoacylation activity of purified ERS proteins. Aminoacylation reactions were performed with 100 nM MBP-ERS_RC_ (orange) or MBP-ERS_2.5W_ (blue) and 4 µM tRNA^Glu^ under the conditions described in Experimental Procedures. |
| --- |

**
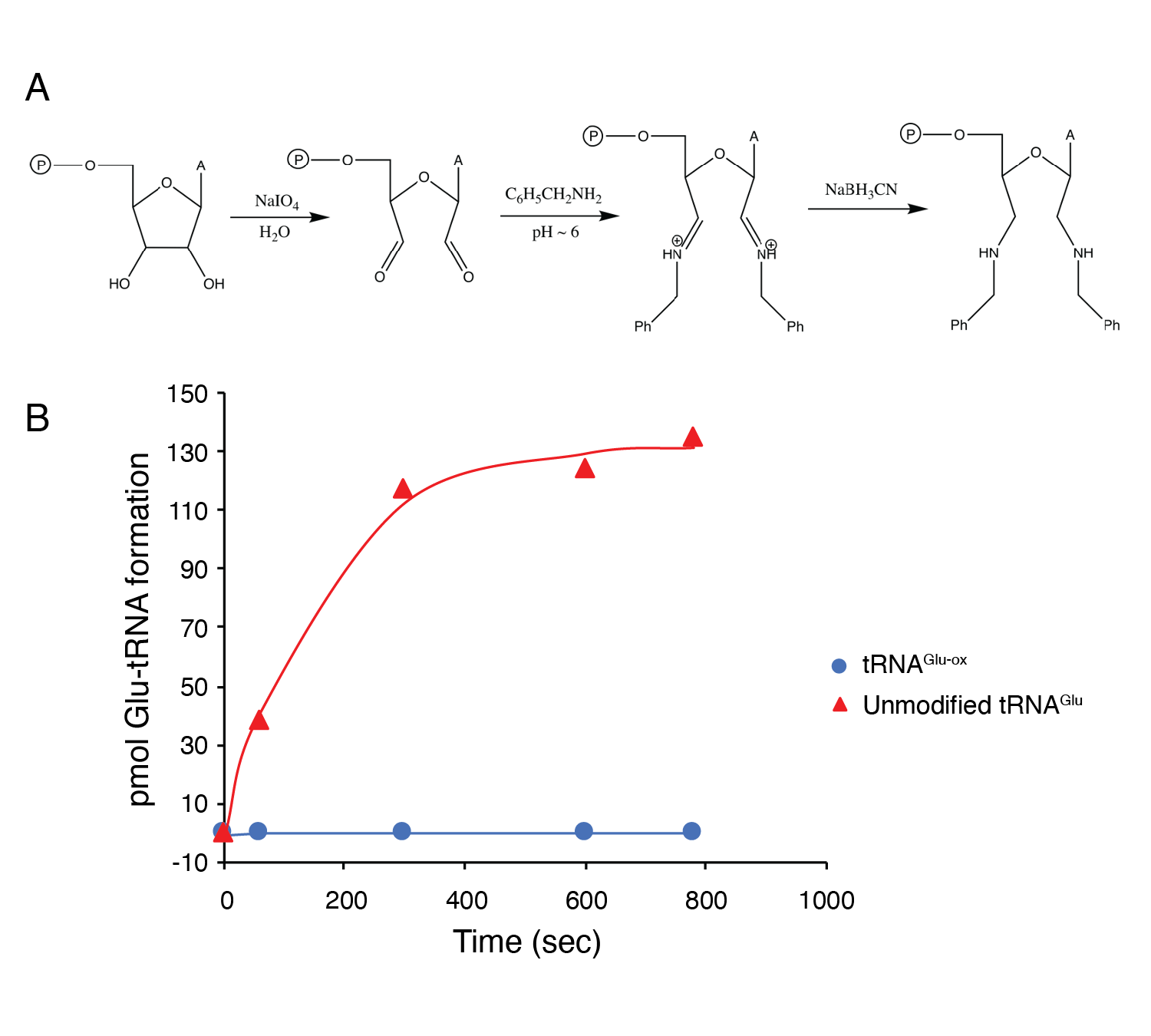
**

| Figure S4. 3′-end modification of *in vitro* transcribed tRNA^Glu(TTC)^. (A) Scheme of reaction at the ribose ring of the tRNA 3′ adenosine. *In vitro* transcribed tRNA was oxidized by sodium periodate and stabilized with benzylamine/sodium cyanoborohydrate. (B) Assessing the aminoacylation capability of 3′-end modified tRNA (tRNA^Glu-ox^, blue) compared to unmodified tRNA (red). Aminoacylation reactions were performed with 100 nM MBP-ERS_2.5W_ and 4 µM tRNA under the conditions described in Experimental Procedures. |
| --- |

**
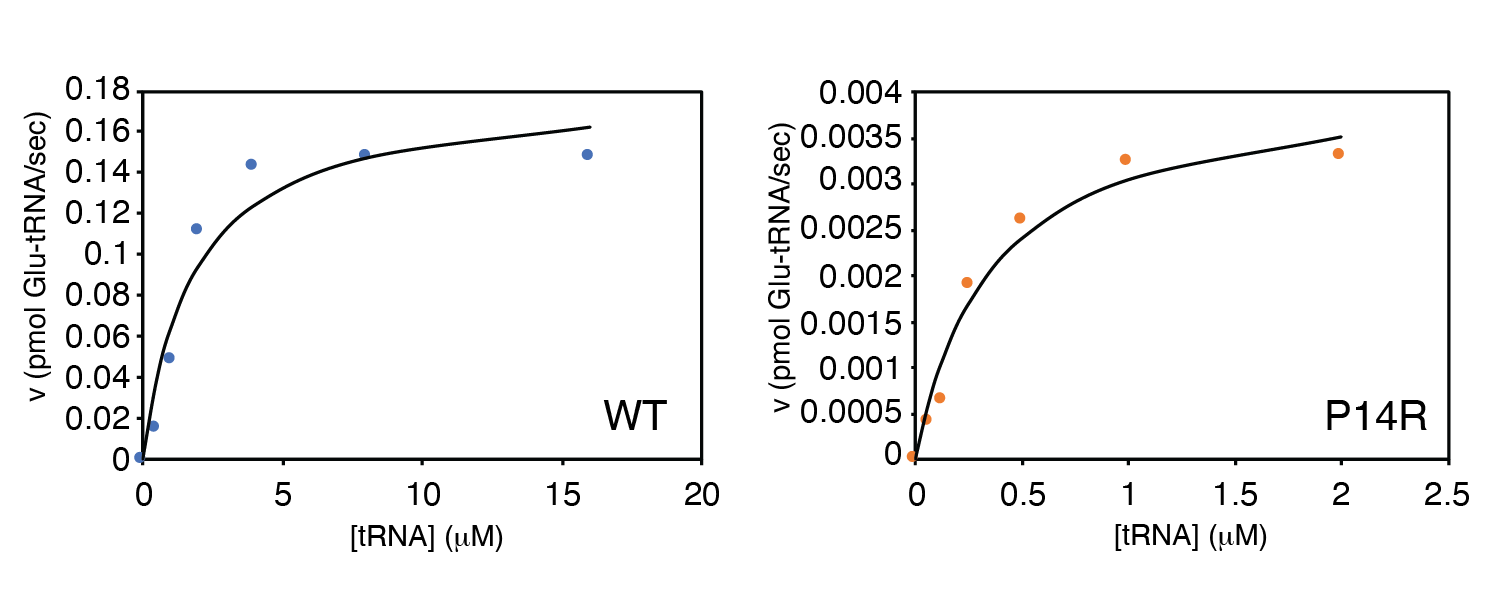
**

| Figure S5. Initial velocity vs. substrate plots for aminoacylation by MBP-ERS_2.5W_ WT and P14R enzymes. Aminoacylation reactions were performed with 100 nM (WT) or 10 nM (P14R) MBP-ERS_2.5W_ enzymes and varying tRNA concentrations. A representative Michaelis-Menten fit of three replicates is shown for each enzyme. Kinetic parameters *k*_cat_ and *K*_M_ were determined and are listed in Table 2. |
| --- |

**
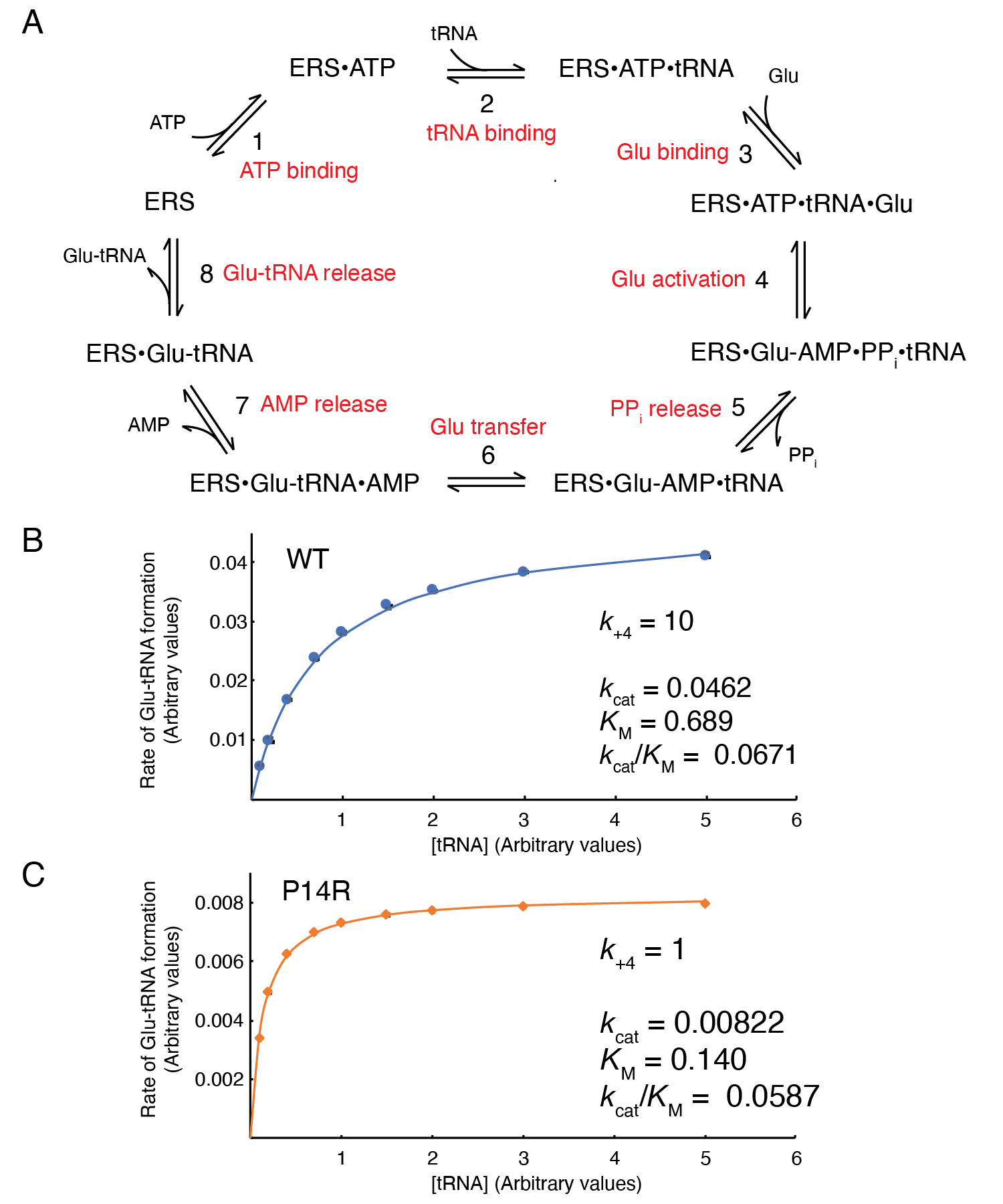
**

| Figure S6. Data simulation for WT and P14R ERS aminoacylation. (A) Multi-step reaction model for ERS aminoacylation. In this model, we assumed tRNA binding is a requirement for Glu activation, which is reversible. It was also assumed that the rate of the Glu activation step k_+4_ is 10-fold higher for WT relative to P14R, and that the rates for steps 1-3, 5-8 are the same for WT and P14R. For P14R, the rate of Glu activation is slower than Glu transfer and is rate-limiting. (B, C). Formation of Glu-tRNA was simulated for WT and P14R using the Global Kinetic Explorer (42). Data were fit to the Michaelis-Menten equation and the *K*_M_ and *k*_cat_ values derived from the fit are listed on the graphs. This simulation predicts that the mutant displays reduced *K*_M_ and *k*_cat_ values relative to WT but a similar overall *k*_cat_/*K*_M_. |
| --- |


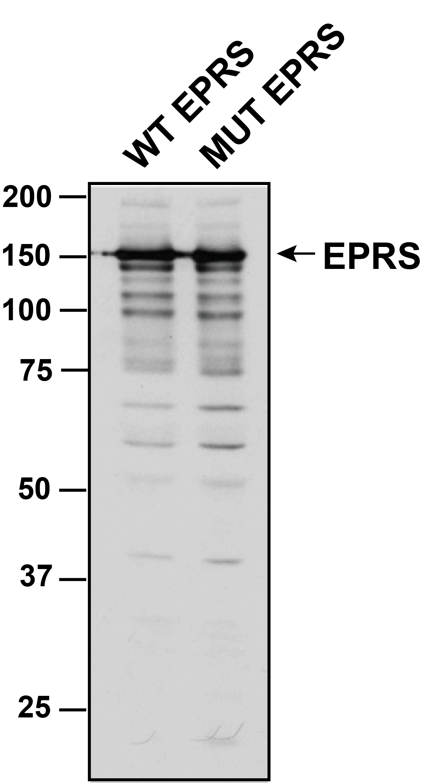


**Figure S7. EPRS protein immunoblotting in patient-derived and WT cell lysates.** WT and patient (MUT) fibroblast cells were cultured in DMEM supplemented with 10% FBS to about 70% confluency. Protein lysates were then prepared from the cells and separated by SDS-PAGE, followed by immunoblotting using PRS-specific antibody. MW markers are shown in kDa.

**
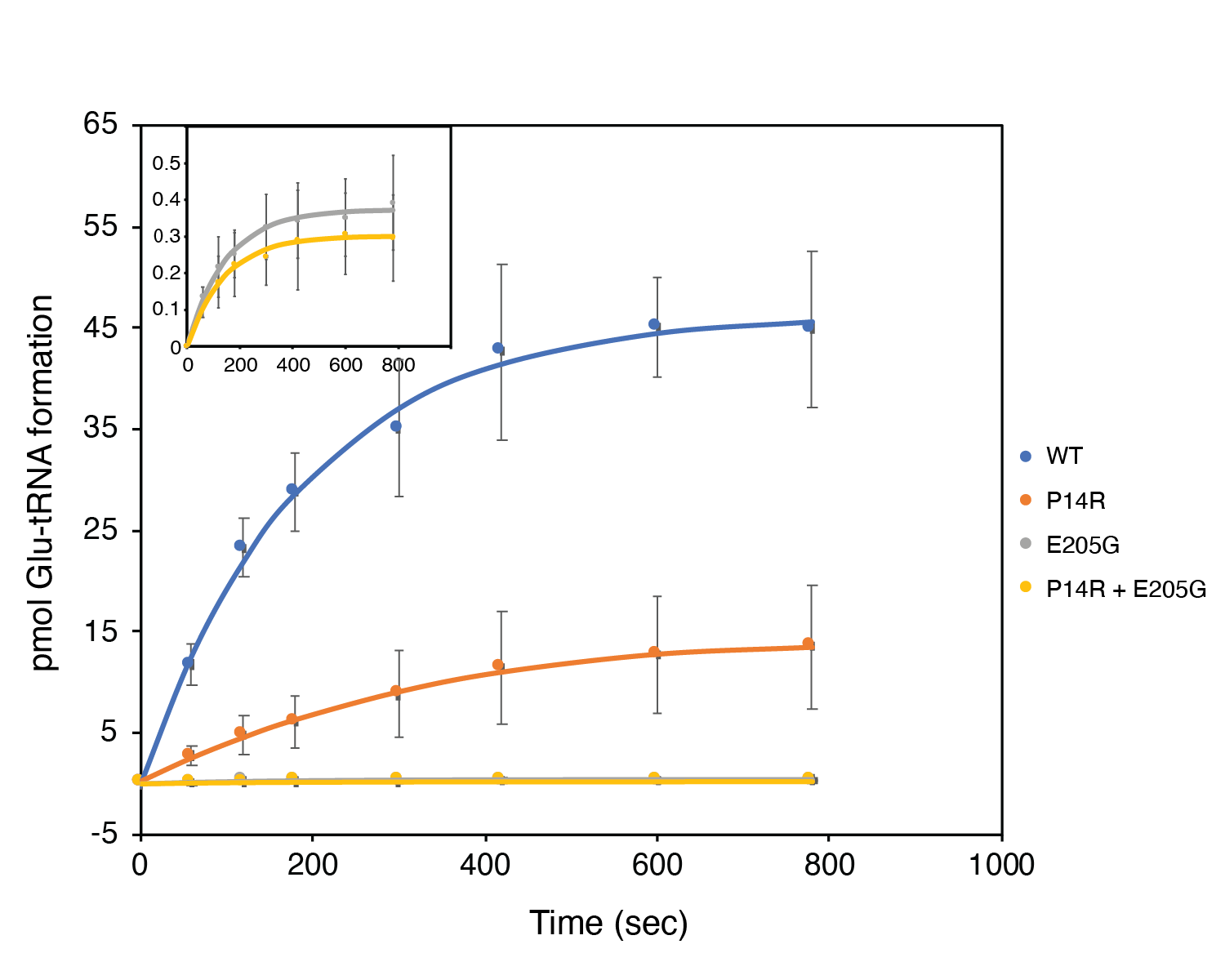
**

Figure S8. Aminoacylation of tRNA^Glu(TTC)^ by WT and mutant MBP-ERS_2.5W_. Aminoacylation reactions were performed with 4 μM tRNA and 100 nM enzymes under the conditions described in Experimental Procedures. The inset shows the enzymes containing the E205G mutation on an expanded Y-axis scale.
